# Magnetic Ordering and Bonding in Ln_3_TiBi_5_ (Ln = Pr, Nd, Gd)

**DOI:** 10.1021/acsmaterialsau.6c00162

**Published:** 2026-08-20

**Authors:** Kaige Wu, Danila S. Sokratov, Ram Kumar, Hope A. Long, Johnpierre Paglione, Vladislav V. Klepov

**Affiliations:** † Department of Chemistry, 1355 University of Georgia, Athens, Georgia 30602, United States; ‡ Maryland Quantum Materials Center and Department of Physics, University of Maryland, College Park, Maryland 20742, United States; § Canadian Institute for Advanced Research, Toronto, Ontario M5G 1Z8, Canada

**Keywords:** hypervalent bonding, topological materials, magnetism, bismuth, lanthanides

## Abstract

Topological materials have gained remarkable attention due to their exotic properties and potential applications in advanced devices. The Ln_3_MPn_5_ (Ln = lanthanide, M = transition metal, Pn = pnictide) family, which hosts hypervalent Bi chains, has been identified as a promising platform to study magnetic topological materials. Herein, we present single-crystal growth of new compounds Ln_3_TiBi_5_ (Ln = Pr, Nd, and Gd) and their property characterization. Magnetic measurements on Pr_3_TiBi_5_ show no apparent indications of magnetic ordering down to 2 K, whereas both Nd_3_TiBi_5_ and Gd_3_TiBi_5_ show antiferromagnetic ordering at 6.2 and ∼18 K, respectively. Gd_3_TiBi_5_ exhibits antiferromagnetic transitions at 17.1 K (*H* ∥ *c*) and 18.8 K (*H* ⊥ *c*), respectively, suggesting competing ground states of this compound. Heat capacity measurements on the samples confirm the presence of magnetic ordering. DFT calculations reveal band crossings that are located above the Fermi level in Pr_3_TiBi_5_ and Nd_3_TiBi_5_, while the band structure of Gd_3_TiBi_5_ exhibits topologically nontrivial states near the Fermi level. In the presence of SOC, the electronic structure of Gd_3_TiBi_5_ changes dramatically. In particular, a local gap opens at the band crossing along M–K, and the band crossing along Γ–A moves above the Fermi level. The crystal orbital Hamilton population (COHP) calculations illustrate the bonding character of Ln-Bi_6g_, Ln-Bi_4d_, Ti–Bi_6g_, Bi_4d_-Bi_4d_, and Ti–Ti in Ln_3_TiBi_5_ (Ln = Pr, Nd, Gd). Ln forms the strongest bonds with Bi_6g_, followed by Ti–Bi_6g_ and Ln-Bi_4d_. Bi_4d_–Bi_4d_ in 1D Bi^2–^ chains forms hypervalent bonding, with both bonding and antibonding states below the Fermi level. Ti–Ti interactions are relatively weak compared with the other bonding interactions. This work fills the gap in the Ln_3_TiBi_5_ family with the synthesis of Pr_3_TiBi_5_ and Nd_3_TiBi_5_ and also extends it to Gd_3_TiBi_5_, the first Gd-containing compound in the anti-Hf_5_Sn_3_Cu-type series.

## Graphical abstract

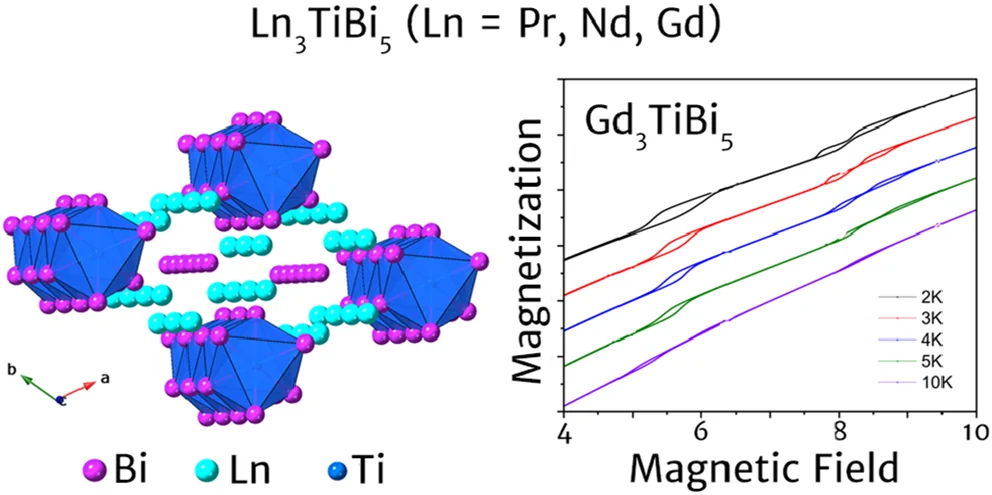

## Introduction

Topological semimetals (TSMs) are a class of gapless quantum materials exhibiting protected band crossing in the bulk band structure and vanished density of states near the Fermi energy.^1–4^ The most common TSMs are Dirac semimetals (DSMs) and Weyl semimetals (WSMs).^5–8^ DSMs possess a Dirac point formed by the crossing of two sets of linearly dispersed bands at a single point.^9^ The two sets of bands are 2-fold degenerate, making a Dirac point 4-fold degenerate.^8^ The breaking of either inversion symmetry or time-reversal symmetry in DSMs results in WSMs,^4,10,11^ which exhibit a crossing of non-degenerate bands.^12–14^ The crossing of linearly dispersed bands in the bulk is a defining feature of TSMs, which leads to the emergence of massless Dirac and Weyl Fermions.^4,15^ Because of the “massless” nature, a distinguished feature of Dirac and Weyl Fermions is their ultrafast mobility,^9,16–19^ giving rise to a series of exotic phenomena such as a huge magnetoresistance effect,^20–22^ quantum Hall effect,^23^ quantum oscillations,^24^ etc. Apart from that, linear band dispersion also induces exotic optical properties like optical conductivity and long-wavelength photon absorption.^9,25,26^ In addition, nonlinear optical effects have been noticeable in WSMs with chiral crystal structures.^27,28^ Due to these distinguishing properties, TSMs hold significant promise in applications for spintronics, catalysis, and optical devices.^29–34^ TSMs opened a pathway to a new class of quantum materials, which has sparked a tremendous interest in predicting and discovering new TSMs among condensed matter physics and solid-state chemistry communities.^35–38^

Despite an abundance of computationally predicted topological materials, their realization remains a challenge due to difficulties in their characterization. While first-principles calculations have been at the forefront of revealing nontrivial topology in the electronic structures of materials,^39–41^ angle-resolved photoemission spectroscopy (ARPES) remains a primary experimental technique in verifying topological phases owing to its unique ability to directly probe the electronic structure with energy and momentum resolution.^42–45^ Recently, Schoop et al. introduced simple chemical design rules that allow for predicting TSM properties in materials from a chemist’s perspective, enabling the synthesis of new topological phases based on structural considerations.^46^ One of the two requirements is that the unit cell of TSM candidate should contain two chemically equivalent atoms, which enables band folding and leads to band inversion and band crossing, a primary source for a topological phase.^46,47^ The second requirement is the presence of extended multicenter hypervalent bonds, where electrons delocalize over a structural motif, enabling its partial electron filling.^46–48^ It was proposed by Hoffmann to explain several networks of heavy p-group elements that are against the Zintl–Klemm electron-precise rule.^48^ The hypervalent bonds can not only stabilize the topological band structure for a half-filled band and thus locate the Fermi level to the crossing point by avoiding Peierls distortion but also enhance the material’s tolerance to defects that would alter the position of Fermi level through the formation of steep and widely dispersed bands.^46,47,49–51^

One motif that follows the chemical rules for TSMs is the 1D Bi^2–^ chain in Ln_3_MPn_5_ (Ln = lanthanide or actinide, M = transition metal, Pn = Sb or Bi) compounds crystallizing in the anti-Hf_5_Sn_3_Cu-type, and it has been identified as a suitable platform to study topological materials.^51^ In this structure type, each chain contains two Pn atoms within the unit cell, enabling band folding.^51^ The seven electrons per Pn^2–^ anion lead to a half-filled p_z_ orbital per Pn atom and electron-rich, multicenter hypervalent bonding.^49,51^ The half-filled orbitals pin the Fermi level at the crossing point in the folded band structures.^51^ The hypervalent bonding stabilizes the half-filled orbitals and thus protects the band structure that would form an energy gap due to the Peierls distortion.^49,51^ A unit cell has two chains, the interaction of which finally brings about a topological band structure.^51^ One example of such compounds is Sm_3_ZrBi_5_, which has been recently identified as a new magnetic topological material. Despite significant synthetic efforts toward the synthesis of new bismuthide materials, there are still gaps in the composition phase space that limit our understanding of the stability ranges and properties in this promising class of materials, necessitating the discovery and single-crystal growth of new members of this family.

To extend the family of potential topological phases, we focus on the Ln_3_TiBi_5_ (Ln = lanthanide) anti-Hf_5_Sn_3_Cu-type family of compounds, in which only Ln = La, Ce, and Sm phases have been reported.^52–55^ The size of lanthanides significantly affects the stability of Ln_3_MPn_5_ phases, with early lanthanides, La^3+^–Sm^3+^, favoring this structure type, whereas the remaining lanthanides tend to form ternaries with other structure types.^49,56–62^ In this report, we not only fill the gap in the Ln_3_TiBi_5_ family by synthesizing Pr_3_TiBi_5_ and Nd_3_TiBi_5_ but also extend it to Gd_3_TiBi_5_, the first Gd-containing compound in the anti-Hf_5_Sn_3_Cu-type structure. The synthesis of high-quality single crystals of three new members in Ln_3_TiBi_5_ (Ln = Pr, Nd, Gd) enables the characterization of their structural and magnetic properties. The experimental data are corroborated by a detailed computational study that reveals the features of their electronic structures.

## Results and Discussion

### Crystal Growth and Crystal Structure

Large, mm-scale crystals of Ln_3_TiBi_5_ (Ln = Pr, Nd, and Gd; Figure 1c–e) were grown via self-flux reactions where Ln and Ti were reacted in the molar ratio of 3:1 and excess Bi was employed as flux.^63–65^ For Ln_3_TiBi_5_ (Ln = Pr, Nd), the reaction temperature was 900 °C. In the synthesis of Gd_3_TiBi_5_, a significant amount of plate-like Kagome phase GdTi_3_Bi_4_ ^66^ formed along with Gd_3_TiBi_5_ at 900 °C, so the temperature was set to 750 °C to reduce the amount of the side product.

**Figure 1:**
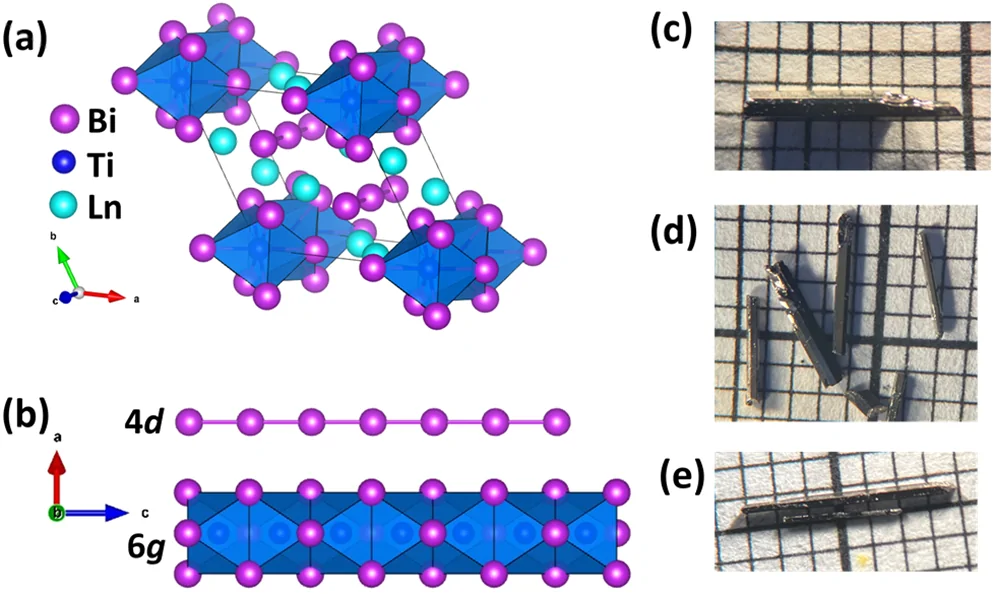
Crystal structure of Ln_3_TiBi_5_ (Ln = Pr, Nd, and Gd). (a) Individual unit cell shown along the c direction. (b) Separate 1D Bi^2–^ motif (top) and 1D TiBi_6_ octahedra motif (bottom). Optical microscope images of (c) Pr_3_TiBi_5_, (d) Nd_3_TiBi_5_, and (e) Gd_3_TiBi_5_, respectively, on a millimeter paper.

As shown in Figure 1 and Table S1, Ln_3_TiBi_5_ (Ln = Pr, Nd, Gd) crystallizes in the space group *P*6_3_/*mcm* and the *anti*-Hf_5_Sn_3_Cu structure type. 1D Bi^2–^ chains and 1D face-sharing TiBi_6_ octahedra run along the *c* axis, charge balanced by Ln^3+^ (Ln = Pr, Nd, Gd). Bi atoms occupy two different Wyckoff positions; 4d positions contain Bi^2–^ chain atoms, and the 6g Bi atoms form TiBi_6_ octahedra (Figure 1b).

To better understand the structure of this family, we list the bond lengths of Bi–Bi and M-Bi in Ln_3_MBi_5_ (Ln = La, Ce, Pr, Nd, Sm, Gd; M = Sc, Ti, Mn, Zr, Hf, Mg)^49,51,52,58,67–69^ in Table S2. All the Bi–Bi bond lengths in the chains lie within a 3.17–3.28 Å range and are longer than elemental Bi (∼3.07 Å), suggesting the existence of hypervalent bonds.^51–53^ Seven electrons per atom in the Bi^2–^ chains lead to electron-rich multicenter bonding (hypervalent bonding).^48,49,51,52^ Each Bi atom in Bi^2–^ chains contains filled s, p_*x*_, p_*y*_ orbitals, and a half-filled p_*z*_ orbital.^49,51^ The half-filled p_*z*_ orbitals bring about one electron each to the hypervalent bond, causing longer Bi–Bi lengths than two-center two-electron bonds, which have more localized interactions.^47–49,51^ With the same M in Ln_3_MBi_5_, Bi–Bi and M-Bi bond lengths shorten as the radius of lanthanides becomes smaller (Figure S1a). For example, Bi–Bi and M-Bi bond lengths reduce from 3.2010 and 2.9301 Å in Pr_3_TiBi_5_ to 3.1719 and 2.9234 Å in Gd_3_TiBi_5_, respectively, which agrees with lanthanide contraction. Similarly, a comparison of the Bi–Bi and M-Bi bond lengths in Ln_3_MBi_5_ (M = Ti, Zr, Hf) with the same Ln also shows the expected trends due to secondary periodicity due to lanthanide contraction (Figure S1a).^49^ The bond lengths in Ln_3_TiBi_5_ are shorter than those in Ln_3_ZrBi_5_ as Zr has a larger radius (Figure S1), but the transition to Ln_3_HfBi_5_ results in only slightly longer bonds. A similar trend is found in Ln_3_MSb_5_ with the same Ln (Ln = La, Ce, Pr, Nd, Sm; M = Ti, Zr, Hf),^62^ in which Ln_3_ZrSb_5_ has the longest bond lengths of Sb–Sb and M-Sb, followed by Ln_3_HfSb_5_ and Ln_3_TiSb_5_ (Figure S1b and Table S3), except for the Sb–Sb bond length in La_3_TiSb_5_.

The size of lanthanides significantly affects the stability of Ln_3_MPn_5_ phases.^49^ Khoury et al. proposed a comprehensive diagram of phase stability in this family by collating a structural map of known Ln_3_MPn_5_ compounds and found that early lanthanides, La^3+^–Sm^3+^, and U^3+^ favor the Ln_3_MPn_5_ composition, whereas lanthanides smaller than Sm^3+^ tend to form ternaries with other structure types.^49^ Although Pr_3_TiBi_5_ and Nd_3_TiBi_5_ fall well within the proposed stability range of the Ln_3_MPn_5_ type, the synthesis of Gd_3_TiBi_5_ suggests that more compounds with smaller lanthanide atoms can be obtained, further extending this structure type.

### Electronic Structure

The band structures of Pr_3_TiBi_5_ and Nd_3_TiBi_5_ calculated without spin–orbit coupling (SOC) exhibit band crossings above the Fermi level along the Γ–A path (Figures S2 and S3). Gd_3_TiBi_5_, without SOC (Figure 2a), features topological band crossing^51^ near the Fermi level along M–K and Γ–A high-symmetry lines. The two band crossings, however, stem from distinct contributions of p orbitals at the 4d and 6g Wyckoff positions of Bi, which is revealed by orbital-projected band structures for Gd_3_TiBi_5_ (Figures S4–S6). The band crossing near the Fermi level along the M-K path is mainly derived from p_*x*_ and p_*y*_ orbitals of Bi at the 6g Wyckoff position, with significantly weaker contributions from p_*x*_ and p_*y*_ orbitals of Bi at the 4d Wyckoff position and no involvement of the p_*z*_ orbital from either site. In contrast, the band crossing near the Fermi level along the Γ–A direction is predominantly contributed by p orbitals of Bi at the 4d Wyckoff position, in which the p_*z*_ orbital plays the leading role. A smaller contribution comes from p_*x*_ and p_*y*_ orbitals of Bi at 6g and 4d Wyckoff positions. This analysis indicates that Bi atoms in both the Bi–Bi chains and TiBi_6_ octahedra play a crucial role in establishing the overall topological character of the band structure. The band crossing along Γ–A exhibits a steeper dispersion than that along the M-K direction, indicating greater robustness to SOC-induced gaps. This is demonstrated by the band structure of Gd_3_TiBi_5_ with SOC (Figure 2c), where a local gap of around 0.044 eV opens along the M-K direction. While the local energy gap is smaller than that of most known topological insulators, it remains significant.^51^ In contrast, SOC induces no local gap for the band crossing along Γ–A. Instead, it shifts the band crossing upward to approximately 0.44 eV above the Fermi level. Additionally, SOC shifts the degeneracy at the A point closer to the Fermi level, and a degeneracy at the L point emerges near the Fermi level.

**Figure 2:**
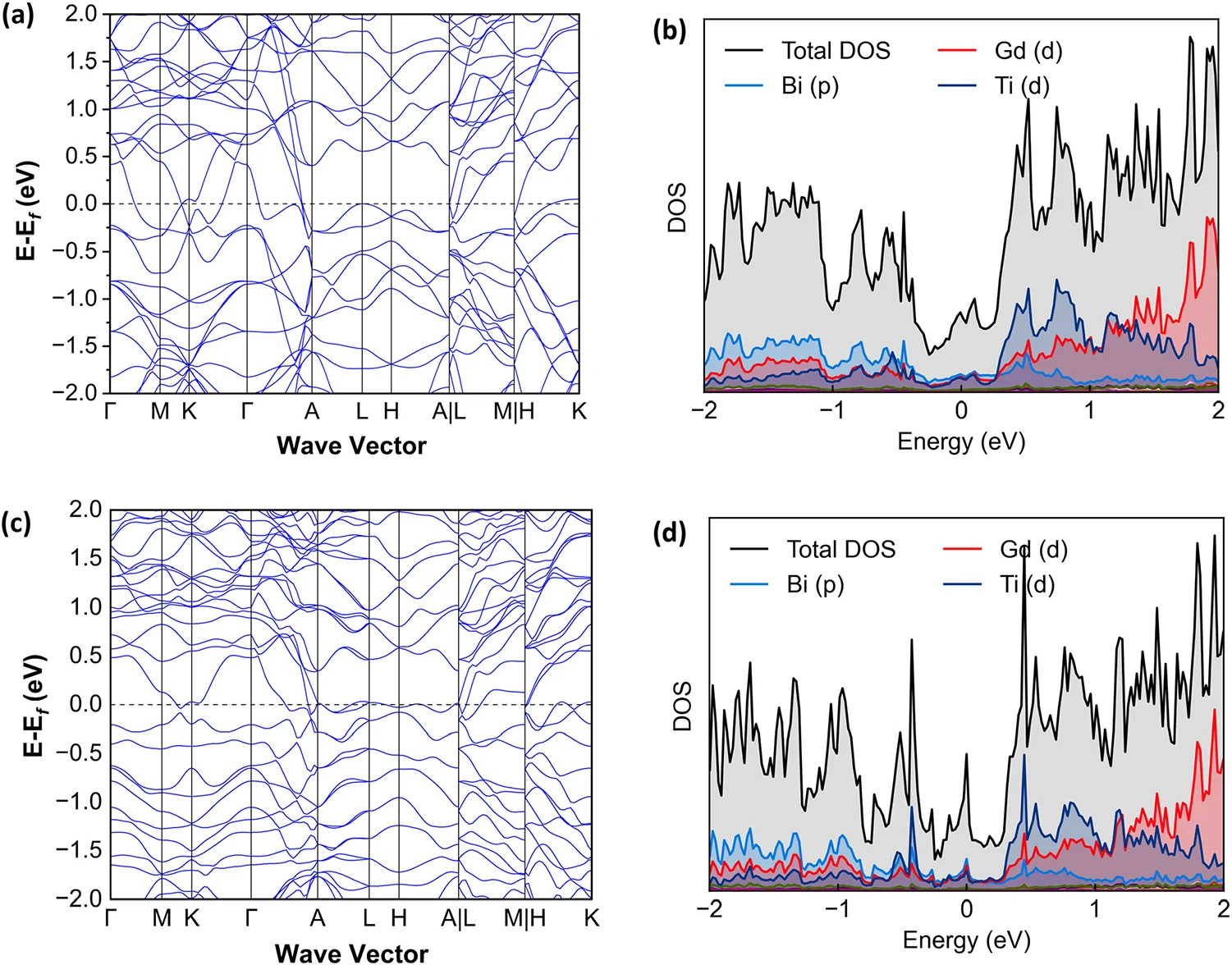
Electronic structure of Gd_3_TiBi_5_. (a) Band structure of Gd_3_TiBi_5_ without spin–orbit coupling. (b) Density of states of Gd_3_TiBi_5_ without spin–orbit coupling. (c) Band structure of Gd_3_TiBi_5_ with spin–orbit coupling. (d) Density of states of Gd_3_TiBi_5_ with spin–orbit coupling.

Both the total and partial density of states (DOS), without and with SOC (Figure 2b,d), show that Bi p, Ti d, and Gd d orbitals contribute the most to the Fermi level. Furthermore, the DOS peaks with SOC are sharper than those without SOC, which can be attributed to SOC-induced band flattening. As observed in the band structure (Figure 2c), SOC reduces band dispersion, resulting in flatter bands and consequently a higher density of states at the Fermi level (Figure 2d). A higher density of states can enhance electron–electron interactions, leading to stronger electronic correlations, which may give rise to the emergence of exotic physical phenomena.^70^ Overall, the electronic behavior of Gd_3_TiBi_5_ is similar to Sm_3_ZrBi_5_.^51^

To get insights into the bonding features of Ln_3_TiBi_5_ (Ln = Pr, Nd, Gd), we calculated the crystal orbital Hamilton population (COHP). The optimized bond distances and integrated −COHP (–ICOHP) are listed in Table 1. As shown in Figures S7–S9, the interactions of Ln and Bi at the 6g Wyckoff position (Pr–Bi_6g_, Nd–Bi_6g_, Gd–Bi_6g_) are strongly bonding, with all bonding status below the Fermi level and –ICOHP of 2.21, 2.21, and 1.81 eV/bond, respectively. The optimized distances of Ln to Bi at the 4g Wyckoff position (Pr–Bi_4d_, Nd–Bi_4d_, Gd–Bi_4d_) are 3.448, 3.431, and 3.380 Å, respectively, longer than that of corresponding Ln-Bi_6g_ (3.272, 3.256, and 3.212 Å), resulting in weaker but still strongly bonding with –ICOHP of 1.52, 1.53, and 1.27 eV/bond, respectively. Ln-Bi_4d_ (Ln = Pr, Nd, Gd) interactions are bonding below the Fermi level (Figures S7–S9). The Ti–Bi_6g_ interactions are strongly bonding, with -ICOHP values of 1.64, 1.64, and 1.60 eV/bond, respectively. They also show predominant bonding below the Fermi level (Figures S7–S9). The COHP curve of Bi_4d_-Bi_4d_ (Figures S7–S9) within the 1D Bi chains in Ln_3_TiBi_5_ exhibits both bonding and antibonding below the Fermi level, which is characteristic of hypervalent interactions in metal pnictides.^71^ The Bi_4d_-Bi_4d_ interactions are overall bonding, as evidenced by the −ICOHP values of 1.26, 1.26, and 1.25 eV/bond, respectively. The Ti–Ti interactions in Ln_3_TiBi_5_ are weaker than other interactions, with -ICOHP values of 0.65, 0.67, and 0.70 eV/bond, respectively, but still strongly bonding with a predominant bonding status below the Fermi level (Figures S7–S9).

**Table 1:** Selected Interactions along with Optimized Bond Distances and Corresponding –ICOHP of Ln_3_TiBi_5_ (Ln = Pr, Nd, Gd)

compound	interaction	distance (Å)	–ICOHP per bond (eV)
Pr_3_TiBi_5_	Pr–Bi_6g_	3.272	2.21
	Pr–Bi_4d_	3.448	1.52
	Ti–Bi_6g_	2.961	1.64
	Bi_4d_-Bi_4d_	3.232	1.26
	Ti–Ti	3.232	0.65
Nd_3_TiBi_5_	Nd–Bi_6g_	3.256	2.21
	Nd–Bi_4d_	3.431	1.53
	Ti–Bi_6g_	2.958	1.64
	Bi_4d_-Bi_4d_	3.222	1.26
	Ti–Ti	3.222	0.67
Gd_3_TiBi_5_	Gd–Bi_6g_	3.212	1.81
	Gd–Bi_4d_	3.380	1.27
	Ti–Bi_6g_	2.947	1.60
	Bi_4d_-Bi_4d_	3.194	1.25
	Ti–Ti	3.194	0.70

### Magnetic Properties

The temperature dependence of the magnetic susceptibility and inverse magnetic susceptibility with the magnetic field applied parallel and perpendicular to the *c* axis of single crystals of Ln_3_TiBi_5_ (Ln = Pr, Nd, Gd) is shown in Figure 3. Pr_3_TiBi_5_ shows no magnetic transitions for both field orientations (*H* ⊥ *c* and *H* ∥ *c*), though we do note there is a broad hump-like feature that can be seen near 30 K (Figure 3a), possibly an indication of a competition between ferromagnetic (FM) and antiferromagnetic (AFM) correlations within the compound. This observation is more pronounced in inverse magnetic susceptibility 1/χ versus temperature plots (Figure 3b). The Curie–Weiss fits of the data from 120 to 300 K yield Curie–Weiss temperatures of θ_C–W,∥_ = −61.62 K and θ_C–W,⊥_ = 16.17 K, and effective magnetic moments of μ_∥_ = 3.27 and μ_⊥_ = 3.41 μ_B_, when the field is applied parallel and perpendicular to the *c* axis of the Pr_3_TiBi_5_ crystal, respectively. The easy axis is in the ab-plane. An average effective magnetic moment that is calculated as μ_av_ = (μ_∥_ + 2 μ_⊥_)/3 is equal to 3.36 μ_B_, which agrees well with the calculated value of 3.58 μ_B_ for Pr^3+^. For Nd_3_TiBi_5_, magnetic susceptibility χ_⊥_(*T*) with the magnetic field applied perpendicular to the *c* axis (*H* ⊥ *c*) of the crystal exhibits paramagnetic behavior between 6.3 and 300 K (Figure 3c). The susceptibility peaks at a Néel temperature (*T*_N_) of 6.2 K and drops rapidly at lower temperatures, indicating an antiferromagnetic transition. The χ_∥_(*T*) data with the magnetic field applied parallel to the *c* axis of the crystal (*H* ∥ *c*) also show an antiferromagnetic ordering at 6.2 K but exhibit an overall lower magnetic susceptibility (Figure 3c). The crystal displays a significant magnetocrystalline anisotropy with χ_⊥_(*T*_N_)/χ_∥_(*T*_N_) = 3.45. Curie–Weiss law fits of the inverse magnetic susceptibility (Figure 3d) perpendicular and parallel to the *c* axis, χ_⊥_^–1^(*T*) and χ_∥_^–1^(*T*), from 120 to 300 K result in effective magnetic moments of 3.60 and 3.57 μ_B_, respectively. The average effective magnetic moment calculated as μ_av_ = (μ_∥_ + 2 μ_⊥_)/3 is equal to 3.59 μ_B_, agreeing with the calculated μ_eff_ of 3.61 μ_B_. The Curie–Weiss temperatures from the fits are θ_C–W,∥_ = −38.13 K and θ_C–W,⊥_ = 4.31 K. Gd_3_TiBi_5_ exhibits antiferromagnetic transitions at 17.1 K (*H* ∥ *c*) and 18.8 K (*H* ⊥ *c*) (Figure 3e). The separate ordering temperatures are unexpected given the fact that the crystal structure has one Gd site, suggesting competing ground states for this compound. There is a notable lack of magnetocrystalline anisotropy in Gd_3_TiBi_5_. One explanation has to do with spin–orbit coupling (SOC) interactions of the rare-earth ions. In a free-standing Gd^3+^ cation, the total orbital angular momentum is 0. This suggests there should be no magnetic anisotropy between *H* ⊥ *c* and *H* ∥ *c* in Gd_3_TiBi_5_. Nonetheless, magnetocrystalline anisotropy could result from crystal structure effects given the quasi-1D structure. The heat capacity data shown in Figure 5a for the Gd_3_TiBi_5_ sample show two clear transitions that coincide with the transition temperatures seen in magnetic susceptibility. This effect could originate from competing ground states near each other in energy, or perhaps from preferential magnetic ordering depending on the applied field orientation. The Curie–Weiss temperatures are θ_C–W,∥_ = −62.39 K and θ_C–W,⊥_ = −61.29 K, obtained by fitting the inverse magnetic susceptibility vs temperature plots (Figure 3f) with the Curie–Weiss law from 120 to 300 K. The effective magnetic moments are μ_∥_ = 8.22 and μ_⊥_ = 8.06 μ_B_. The average effective magnetic moment calculated as μ_av_ = (μ_∥_ + 2 μ_⊥_)/3 is equal to 8.11 μ_B_.

**Figure 3:**
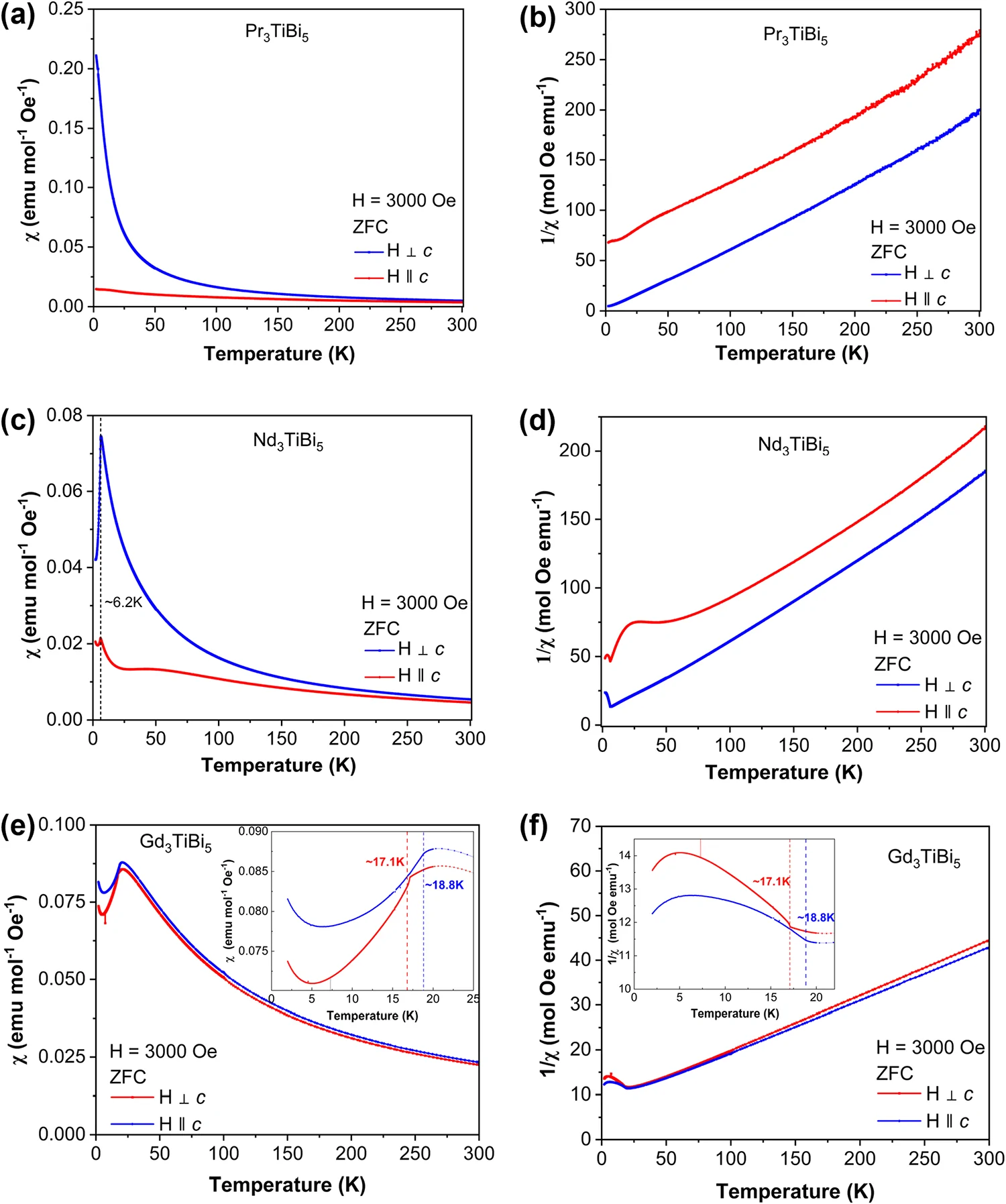
Magnetic susceptibility vs temperature plots of (a) Pr_3_TiBi_5_, (c) Nd_3_TiBi_5_, and (e) Gd_3_TiBi_5_. Inverse magnetic susceptibility vs temperature plots of (b) Pr_3_TiBi_5_, (d) Nd_3_TiBi_5_, and (f) Gd_3_TiBi_5_.

Magnetization vs magnetic field plots of Pr_3_TiBi_5_ show no features within the magnetic fields of 0–7 T and temperatures of 2, 5, and 10 K, though we note that the moment per Pr for *H* ⊥ *c* does not saturate by 7 T (Figure 4a). The Nd_3_TiBi_5_ sample shows the presence of a hysteretic metamagnetic transition near 6.2 T (*H* ⊥ *c*) at 2 K, as well as another metamagnetic transition indicated by the change in slope near 7.2 T (Figure 4b). A similar change in slope of magnetization can be observed around 11.4 T for *H* ∥ *c* at 5 K, which disappears at 2 K (Figure S10). We observe the presence of metamagnetic hysteretic transitions in Gd_3_TiBi_5_. For *H* ⊥ *c*, these hysteretic loops are observed at 2 K between 1 and 3 T, and with increasing temperature the loops shrink until they can no longer be observed at 15 K (Figure 4c). When the field is applied parallel to the *c* axis of the crystal, Gd_3_TiBi_5_ displays metamagnetic transitions near 4–6 T from 2 to 10 K (Figure 4d). Additionally, there are hysteretic metamagnetic transitions between 7.9 and 9 T for *H* ∥ *c*, which morph into one another at a base temperature of 2 K and proceed to separate and close in the middle as the temperature is increased to 3, 4, and 5 K (Figure 4d). Hints of the lower hysteretic transition persist to 10 K (Figure 4d). The exact nature of these metamagnetic transitions is unclear from these data and warrants further study by diffraction techniques. During the preparation of our revised manuscript, another report on Gd_3_TiBi_5_ has been published,^72^ and there is overall agreement between magnetic measurements.

**Figure 4:**
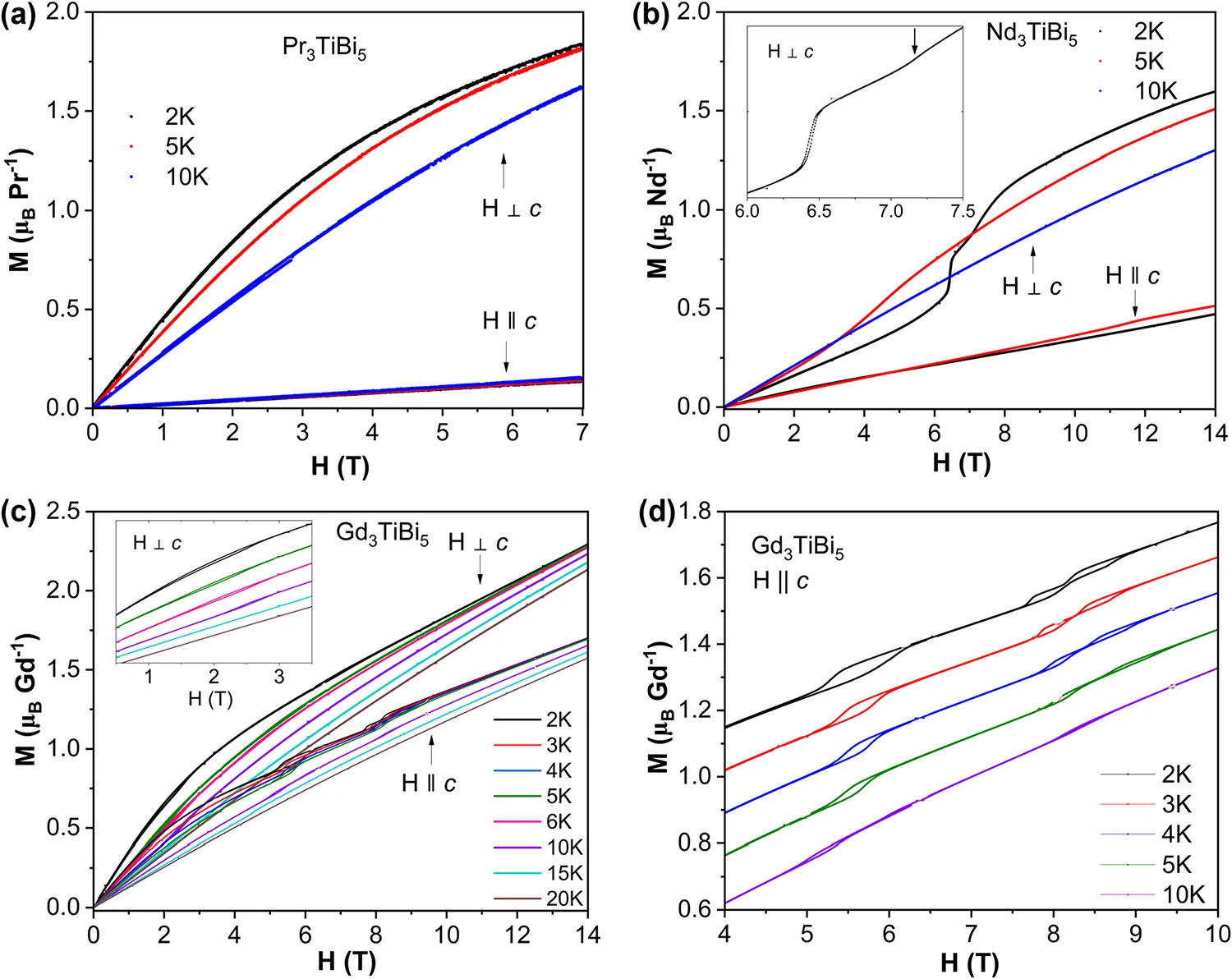
Magnetization vs magnetic field (0–7 T) plots of (a) Pr_3_TiBi_5_, (b) Nd_3_TiBi_5_, and (c) Gd_3_TiBi_5_. Magnetization vs magnetic field (4–10 T) plots of (d) Gd_3_TiBi_5_.

The magnetic properties of Ln_3_TiBi_5_ (Ln = Ce, Pr, Nd, Sm, Gd)^54,55,72^ are summarized in Table S4. Ln_3_TiBi_5_ (Ln = Ce, Nd, Gd) shows antiferromagnetic ordering with transition temperatures of 5.0, 6.2, and 17.1–19 K. Pr_3_TiBi_5_ shows no magnetic ordering down to 2 K, while Sm_3_TiBi_5_ shows an anomaly at 14.8 K with an ordering state different from the conventional antiferromagnetic ordering. Since magnetic exchange in these phases is likely mediated by RKKY interactions, we correlated the de Gennes parameters^73^ for the corresponding lanthanides with magnetic ordering temperatures (Figure S11). While Ln_3_TiBi_5_ (Ln = Ce, Nd, Gd) ordering temperatures follow a linear trend, Pr_3_TiBi_5_ unexpectedly lacks magnetic ordering down to 2 K. On the other hand, the ordering temperature in Sm_3_TiBi_5_, 14.8 K, is significantly higher than expected from the linear trend, ∼8 K. The exact nature of the transition in Sm_3_TiBi_5_ is unknown and can also be attributed to a structural transition or a valence transition, offering an explanation for such a large deviation from the expected value.

### Heat Capacity

Heat capacity (*C*_p_) measurements were performed to further verify the magnetic properties of Ln_3_TiBi_5_ (Ln = Pr, Nd, and Gd). As shown in Figure 5a, Ln_3_TiBi_5_ (Ln = Pr, Nd, Gd) displays a Schottky-like broad feature from 2 to 25 K, which is likely attributed to crystal electric field splitting effects of the rare-earth ions. Pr_3_TiBi_5_ shows no transitions, whereas Nd_3_TiBi_5_ peaks at 5.9 K, consistent with the AFM ordering in the magnetic properties. Two distinct transitions appear at 18.5 and 17.0 K in Gd_3_TiBi_5_, matching the AFM ordering identified from magnetic susceptibility data.

**Figure 5:**
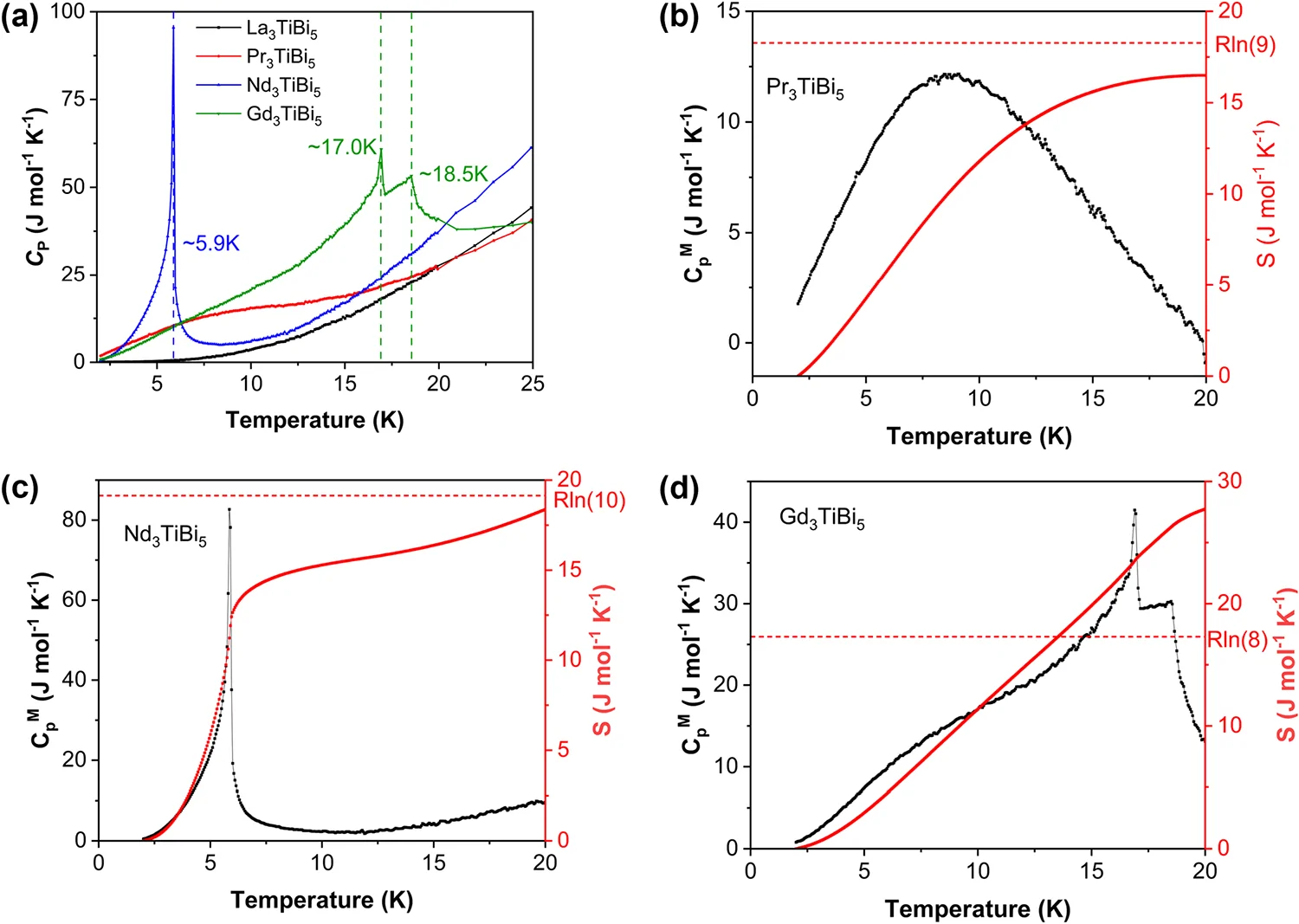
(a) Heat capacity (*C*_p_) of Ln_3_TiBi_5_ (Ln = La, Pr, Nd, Gd); magnetic heat capacity and entropy of (b) Pr_3_TiBi_5_, (c) Nd_3_TiBi_5_, and (d) Gd_3_TiBi_5_. Theoretical maximum entropies are shown as dashed red lines calculated by *S*_max_ = *R *ln­(2*J* + 1). The experimental *S*_m_ were obtained by integrating the magnetic heat capacity, which is shown as red solid curves.

To obtain the magnetic heat capacity contribution of Pr, Nd, and Gd, the heat capacity of La_3_TiBi_5_ was measured and subtracted from that of Ln_3_TiBi_5_ (Ln = Pr, Nd, Gd) down to a base temperature of 2 K. The resulting magnetic heat capacity *C*_p_^M^ and entropy *S*_m_ are shown in Figure 5b–d. The theoretical maximum entropies of Ln_3_TiBi_5_ (Ln = Pr, Nd, Gd), shown as dashed red lines, were calculated by *S*_max_ = *R* ln­(2*J* + 1) where *R* is the ideal gas constant and *J* is the total angular momentum quantum number. *S*_max_ values of Ln_3_TiBi_5_ (Ln = Pr, Nd, Gd) are 18.27, 19.14, and 17.29 J mol^–1^ K^–1^, respectively. The experimental *S*_m_ values were obtained by integrating the magnetic heat capacity, which is shown as red solid curves. The experimental *S*_m_ values of Pr_3_TiBi_5_ and Nd_3_TiBi_5_ are slightly smaller than the theoretical *S*_max_ value, matching up well with the expectations. The experimental *S*_m_ of Gd_3_TiBi_5_ surpasses the theoretical limit, suggesting that the phonon background for the Gd compound could be more complex than that of the nonmagnetic La analogue, though the exact nature of the difference is not understood from this study.

## Conclusions

In this report, we have prepared and studied the properties of Ln_3_TiBi_5_ (Ln = Pr, Nd, and Gd) hosting 1D Bi chains. DFT calculations studied the topological states of Ln_3_TiBi_5_ (Ln = Pr, Nd, Gd), in which band crossing is located above the Fermi level in Pr_3_TiBi_5_ and Nd_3_TiBi_5_, while the band structure of Gd_3_TiBi_5_ exhibits topologically nontrivial regions near the Fermi level without SOC. Since the band structure of Gd_3_TiBi_5_ is primarily derived from Bi, SOC has a strong influence on the band structure, inducing a gap for the band crossing along M-K, moving the band crossing along M-K upward away from the Fermi level, shifting degeneracies toward the Fermi level, and flattening the band structure. COHP calculations indicate the bonding status of Ln-Bi_6g_, Ln-Bi_4d_, Ti–Bi_6g_, Bi_4d_-Bi_4d_, and Ti–Ti in Ln_3_TiBi_5_ (Ln = Pr, Nd, Gd). The bonding and antibonding status below the Fermi level in 1D Bi chains further demonstrates the formation of hypervalent bonding. Magnetic measurements from single crystals show strong magnetocrystalline anisotropy with an easy axis in the *ab*-plane for Pr_3_TiBi_5_ and Nd_3_TiBi_5_ and a much weaker anisotropy in Gd_3_TiBi_5_. Pr_3_TiBi_5_ does not show any ordering, while Nd_3_TiBi_5_ and Gd_3_TiBi_5_ display antiferromagnetic ordering at low temperatures, which was further verified by heat capacity. This work fills the gap in the Ln_3_TiBi_5_ family by synthesizing Pr_3_TiBi_5_ and Nd_3_TiBi_5_ and extends this series to Gd_3_TiBi_5_, a promising topological material.

## Experimental Methods

### Reagents

Bi (Unique Metals, 99.99%) and Ti (Bean Town Chemical, 99.5%) were used as received. La (Luciteria, 99.5%), Pr (Luciteria, 99.6%), Nd (Luciteria, 99.5%), Sm (Luciteria, 99.95%), and Gd (Luciteria, 99.95%) were arc-melted to remove the oxide layers, followed by storage in a glovebox and cutting into smaller pieces before use in a reaction.

### Synthesis

Single crystals of Ln_3_TiBi_5_ (Ln = Pr, Nd, and Gd) were grown via Bi flux reaction. For Ln_3_TiBi_5_ (Ln = Pr and Nd), Ln, Ti, and Bi were loaded into alumina crucibles (9 and 6 mm outer and inner diameters, respectively) in the molar ratio of 3:1:45. The crucibles were then loaded into quartz tubes (12 and 10.5 mm outer and inner diameters, 20 cm long). A piece of silica wool was placed above each crucible for product filtration. After that, the tubes were flame-sealed under vacuum and placed into a programmable box furnace. The sealed reactions were heated to 900 °C over 2 h, held there for 8 h, followed by cooling to 500 °C at a rate of 5 °C/h. Upon cooling to 500 °C, the reactions were immediately centrifuged to remove the excess Bi flux. For Gd_3_TiBi_5_, Gd, Ti, and Bi were combined in the molar ratio of 3:1:90. The sealed reaction was heated to 750 °C over 2 h, held there for 8 h, followed by cooling to 450 °C at a rate of 5 °C/h. The resulting single crystals are silver-metallic with a rod-like morphology and are air-sensitive. To compare the Bi–Bi bond lengths in Bi–Bi chains of Ln_3_TiBi_5_ (Ln = La, Ce, Pr, Nd, Sm, Gd), we synthesized Ce_3_TiBi_5_ and Sm_3_TiBi_5_ to get the cif files. For Ce_3_TiBi_5_, the synthesis method is similar to that of Pr_3_TiBi_5_, except that the molar ratio of Ce, Ti, and Bi is 3:1:60 and the cooling rate from 900 to 500 °C is 100 °C/h. Similarly, Sm_3_TiBi_5_ is prepared using the Pr_3_TiBi_5_ procedure except that the reaction was cooled down from 900 to 500 °C in 12 h. To obtain the magnetic heat capacity contribution of Pr, Nd, and Gd, the nonmagnetic La_3_TiBi_5_ was prepared following the procedure described in ref 53, with a slight change. La, Ti, and Bi with the mass of 0.1, 0.0115, and 3.3 g were combined and reacted at 1000 °C for 6 h followed by cooling to 500 °C in 12 h.

### Single-Crystal XRD

Single-crystal XRD experiments (Mo Kα radiation) were performed on a Bruker D8 QUEST diffractometer equipped with a PHOTON 100 CMOS area detector at room temperature. Structures of Ln_3_TiBi_5_ (Ln = Pr, Nd, and Gd) were solved by the intrinsic phasing method (SHELXT^74^ and Olex2 ^75^) and refined by the full-matrix least-squares method against *F* ^2^ in an anisotropic approximation (SHELXL^76^).

### Energy-Dispersive Spectroscopy (EDS)

The compositions of *Ln*_3_TiBi_5_ (*Ln* = Pr, Nd, Sm, Gd) were confirmed by EDS (Figure S12) performed on a Thermo Fisher Scientific Teneo field-emission scanning electron microscope at the Georgia Electron Microscopy facility.

### Calculations

First-principles calculations were performed using density functional theory (DFT) with the Vienna Ab-initio Package (VASP) plane-wave code,^77,78^ generalized gradient approximation of Perdew, Burke, and Ernzerhof (PBE),^79^ and the projector augmented wave (PAW) method.^80,81^ The energy cutoff for the plane-wave expansion was set to 520 eV. The following pseudopotentials are selected for DFT calculations: Pr_3, Nd_3, Gd_3, Bi_d, and Ti_sv. Structure optimization and self-consistent field (SCF) calculations were performed using a Γ-centered 9 × 9 × 8 Monkhorst–Pack mesh. Density of state calculations were done with and without spin–orbit coupling using Γ-centered 15 × 15 × 15 Monkhorst–Pack mesh.^82^ The *k*-paths for band structure calculations were generated using the VASPKIT package.^83^ The COHP calculation was done using a Γ-centered 15 × 15 × 13 Monkhorst–Pack mesh.^82^ The COHP analysis is performed by LOBSTER program^84^ with the basis set of VASPFit2015 and charge spilling of 0.87%, 0.92%, and 0.90% for Pr_3_TiBi_5_, Nd_3_TiBi_5_, and Gd_3_TiBi_5_.

### Magnetism and Heat Capacity

Temperature- and field-dependent magnetic measurements were carried out with a Quantum Design Magnetic Properties Measurement System (MPMS) and the VSM module of a Quantum Design Dynacool Physical Properties Measurement System (PPMS). χ_*dc*_(*T*) measurements were conducted in zero-field-cooled (ZFC) and field-cooled (FC) modes. Consecutive *M*(*H*) scans at fixed temperatures were started in the ZFC mode by first warming the sample well into the paramagnetic (PM) regime around 100 K, oscillating the field to get rid of any remnant field, and then cooling down to the desired temperatures. All magnetic property measurements were performed on samples mounted on a quartz rod with GE varnish covering the sample to avoid decomposition from exposure to air. Samples were measured with the field along the needles’ long axis (*H* ∥ *c*) and perpendicular to the long axis (*H* ⊥ *c*). Heat capacity measurements were carried out with 14 T PPMS and Dynacool PPMS systems. All shown heat capacity measurements were performed at zero applied field.

## Supplementary Material



## Notes

Crystallographic data, bond lengths of Bi–Bi, M-Bi, Sb–Sb, and M-Sb; summary of magnetic properties of Ln_3_TiBi5 (Ln = Ce, Pr, Nd, Sm, Gd), band structures of Pr_3_TiBi_5_ and Nd_3_TiBi_5_ without SOC, orbital-projected band structures for Gd_3_TiBi_5_ without SOC, COHP plots for Ln_3_TiBi5 (Ln = Pr, Nd, Gd), M-H plots of Nd_3_TiBi_5_ at 2 and 5 K, magnetic transition temperatures vs de Gennes factors in Ln_3_TiBi_5_ (Ln = Ce, Nd, Sm, Gd), EDS data of Ln_3_TiBi_5_ (Ln = Pr, Nd, Gd) (PDF)
