# Supplementary material for: Magnetic Ordering and Bonding in Ln3TiBi5 (Ln = Pr, Nd, Gd)
Source: ACS Mater Au. 2026 Aug 20;6(6):1556–65. doi: 10.1021/acsmaterialsau.6c00162 (PMC13760645; doi:10.1021/acsmaterialsau.6c00162)
Supplement: Supplementary file 1 [file mg6c00162_si_001.pdf]

Supporting Information for

Magnetic Ordering and Bonding in Ln_3TiBi_5 (Ln = Pr, Nd, Gd)

Kaige Wu,[§a] Danila S. Sokratov,[§b] Ram Kumar,[b] Hope A. Long,[a] Johnpierre Paglione,[b,c] Vladislav V. Klepov*[a]

[a] Department of Chemistry, University of Georgia, Athens, Georgia, 30602, United States

[b] Maryland Quantum Materials Center and Department of Physics, University of Maryland, College Park, Maryland 20742, USA

[c] Canadian Institute for Advanced Research, Toronto, Ontario M5G 1Z8, Canada

Table S1. Crystallographic data for Ln_3TiBi_5 (Ln = Ce, Pr, Nd, Sm, Gd)

Empirical formula	Ce_3TiBi_5	Pr_3TiBi_5	Nd_3TiBi_5	Sm_3TiBi_5	Gd_3TiBi_5
Formula weight (g/mol)	1513.16	1515.53	1525.52	1543.85	1564.55
Temperature (K)	300.00	299.00	300.00	299.00	299.00
Crystal system	Hexagonal				
Space group	$P6_3/mcm$				
a/Å	9.5999(4)	9.5597(5)	9.5228(4)	9.4661(11)	9.4318(8)
c/Å	6.4179(4)	6.4020(6)	6.3828(5)	6.3658(10)	6.3438(9)
Volume/Å^3	512.22(5)	506.68(7)	501.27(6)	494.00(14)	488.73(11)
Z	2				
Calculated density/(g/cm^3)	9.811	9.934	10.107	10.379	10.632
μ/mm^{-1}	99.239	101.271	103.322	106.907	110.392
F(000)	1222.0	1228.0	1234.0	1246.0	1258.0
Radiation	MoKα (λ = 0.71073)				
2Θ range, °	4.9 to 54.924	4.92 to 54.992	4.94 to 51.892	4.968 to 54.914	4.986 to 52.856
Index ranges	$-12 \leq h \leq 12$	$-12 \leq h \leq 12$	$-11 \leq h \leq 11$	$-12 \leq h \leq 12$	$-11 \leq h \leq 11$
	$-12 \leq k \leq 12$	$-12 \leq k \leq 12$	$-11 \leq k \leq 11$	$-12 \leq k \leq 12$	$-11 \leq k \leq 11$
	$-8 \leq l \leq 8$	$-8 \leq l \leq 7$	$-7 \leq l \leq 7$	$-8 \leq l \leq 7$	$-7 \leq l \leq 7$
Reflections collected	6164	8437	7738	8434	6096
Independent reflections	238 [R_{int} = 0.0787, R_{sigma} = 0.0257]	237 [R_{int} = 0.0866, R_{sigma} = 0.0235]	200 [R_{int} = 0.0806, R_{sigma} = 0.0207]	233 [R_{int} = 0.0961, R_{sigma} = 0.0267]	204 [R_{int} = 0.0937, R_{sigma} = 0.0286]
Data/restraints/parameters	238/0/14	237/0/14	200/0/14	233/0/14	204/0/14
GOOF	1.149	1.182	1.155	1.224	1.179
Final R indexes [I>=2σ (I)]	R_1 = 0.0202, wR_2 = 0.0490	R_1 = 0.0250, wR_2 = 0.0538	R_1 = 0.0254, wR_2 = 0.0582	R_1 = 0.0236, wR_2 = 0.0475	R_1 = 0.0318, wR_2 = 0.0718
Final R indexes [all data]	R_1 = 0.0223, wR_2 = 0.0501	R_1 = 0.0274, wR_2 = 0.0549	R_1 = 0.0259, wR_2 = 0.0587	R_1 = 0.0255, wR_2 = 0.0480	R_1 = 0.0321, wR_2 = 0.0723
Largest diff. peak/hole /e Å^{-3}	1.72/-1.23	2.32/-1.57	2.10/-1.09	2.31/-1.20	2.89/-1.95

Table S2. Bi-Bi and M-Bi bond lengths (Å) of $Ln_3M\mathrm{Bi}_5$ (*Ln* = La, Ce, Pr, Nd, Sm, Gd; *M* = Sc, Ti, Mn, Zr, Hf, Mg).

	T/K	Bi-Bi	M-Bi
La_3ScBi_5[1]	293	3.2796	3.0229
Ce_3ScBi_5[2]	274 (2)	3.2590	N/A
Pr_3ScBi_5[3]	101	3.2410	3.0018
Nd_3ScBi_5[3]	101	3.2270	2.9974
La_3TiBi_5[4]	N/A	3.2323	2.9358
Ce_3TiBi_5	300	3.2090	2.9319
Pr_3TiBi_5	299	3.2010	2.9301
Nd_3TiBi_5	300	3.1914	2.9267
Sm_3TiBi_5	299	3.1829	2.9220
Gd_3TiBi_5	299	3.1719	2.9234
La_3MnBi_5[5]	193	3.2441	2.9828
Ce_3MnBi_5[5]	193	3.2210	2.9719
Pr_3MnBi_5[5]	193	3.2156	2.9671
Nd_3MnBi_5[5]	193	3.2045	2.9627
La_3ZrBi_5[4]	N/A	3.2599	3.0274
Pr_3ZrBi_5[6]	299	3.2297	3.0207
Nd_3ZrBi_5[6]	299	3.2192	3.0194
Sm_3ZrBi_5[6]	299	3.2079	3.0156
La_3HfBi_5[4]	N/A	3.2543	3.0132
Pr_3HfBi_5[6]	299	3.2215	3.0048
Nd_3HfBi_5[6]	299	3.2126	3.0029
Sm_3HfBi_5[7]	299	3.2024	2.9987
La_3MgBi_5[8]	N/A	3.2746	3.0724

Table S3. Sb-Sb and M-Sb bond lengths (Å) of $Ln_3M\mathrm{Sb}_5$ (*Ln* = La, Ce, Pr, Nd, Sm; *M* = Ti, Zr, Hf)[4, 9].

	Sb-Sb	M-Sb
La_3TiSb_5	3.1401	2.8587
La_3TiSb_5[a]	3.2125	2.9990
La_3ZrSb_5	3.1790	3.0376
La_3HfSb_5	3.1780	3.0362
Ce_3TiSb_5	3.1175	2.9874
Ce_3ZrSb_5	3.1590	3.0137
Ce_3HfSb_5	3.1490	3.0058
Pr_3TiSb_5	3.1120	2.9772
Pr_3ZrSb_5	3.1465	2.9975
Pr_3HfSb_5	3.1355	2.9875
Nd_3TiSb_5	3.0980	2.9625
Nd_3ZrSb_5	3.1375	2.9867
Nd_3HfSb_5	3.1305	2.9796
Sm_3TiSb_5	3.0810	2.9401
Sm_3ZrSb_5	3.1170	2.9654
Sm_3HfSb_5	3.1150	2.9640

The cif file of La_3TiSb_5[a] is from reference 4. All the other cif files are from reference 9. We chose La_3TiSb_5[a] for plotting in Figure S1b.

Table S4. Magnetic properties of Ln_3TiBi_5 (Ln = Ce, Pr, Nd, Sm, Gd).

Compound	Sample type measured	Ordering type	Transition temperature (s)	Magnetic anisotropy (easy direction)	μ_{eff} / Ln (μ_B)		Reference
					H ∥ *c*	H ⊥ *c*	
Ce_3TiBi_5	Single crystal	AFM	5.0 K	Yes, *ab*-plane	2.58	2.60	10
Pr_3TiBi_5	Single crystal	No	N/A	Yes, *ab*-plane	3.27	3.41	This work
Nd_3TiBi_5	Single crystal	AFM	6.2 K	Yes, *ab*-plane	3.57	3.60	This work
Sm_3TiBi_5	Single crystal	Anomaly	14.8 K	Yes, *ab*-plane	1.46	2.01 (H ∥ *a*)	11
Gd_3TiBi_5	Single crystal	AFM	17.1 K (H ⊥ *c*)<br>18.8 K (H ∥ *c*)	No	8.22	8.06	This work
Gd_3TiBi_5	Single crystal	AFM	19 K	No	8.31	8.44 (H ∥ *a'*)<br>8.59 (H ∥ *a*)	12

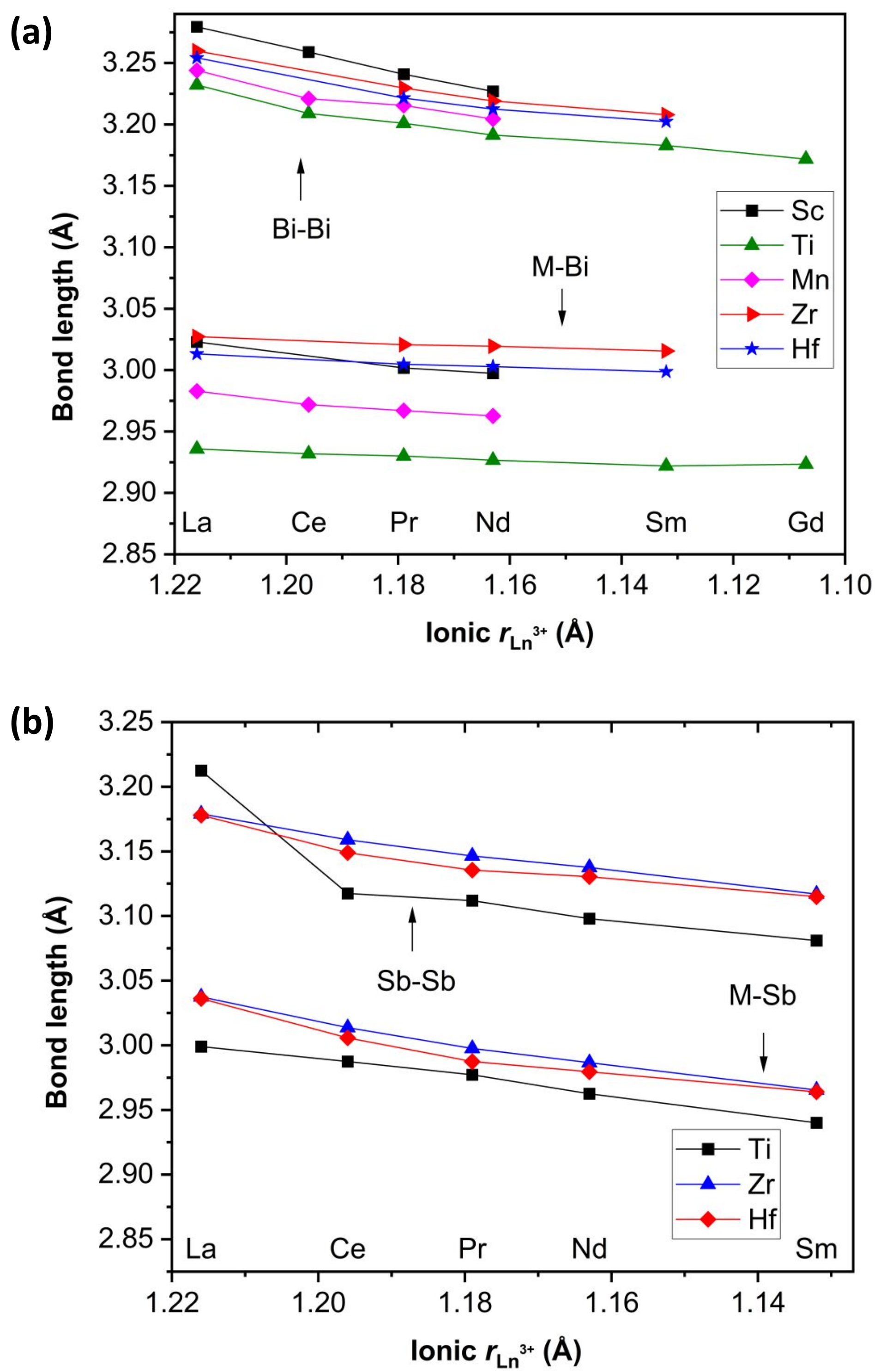

Figure S1. Bond lengths of Bi-Bi and *M*-Bi (*M* = transition metals) in $Ln_3M\text{Bi}_5$ (a), and Sb-Sb and *M*-Sb in $Ln_3M\text{Sb}_5$(b).

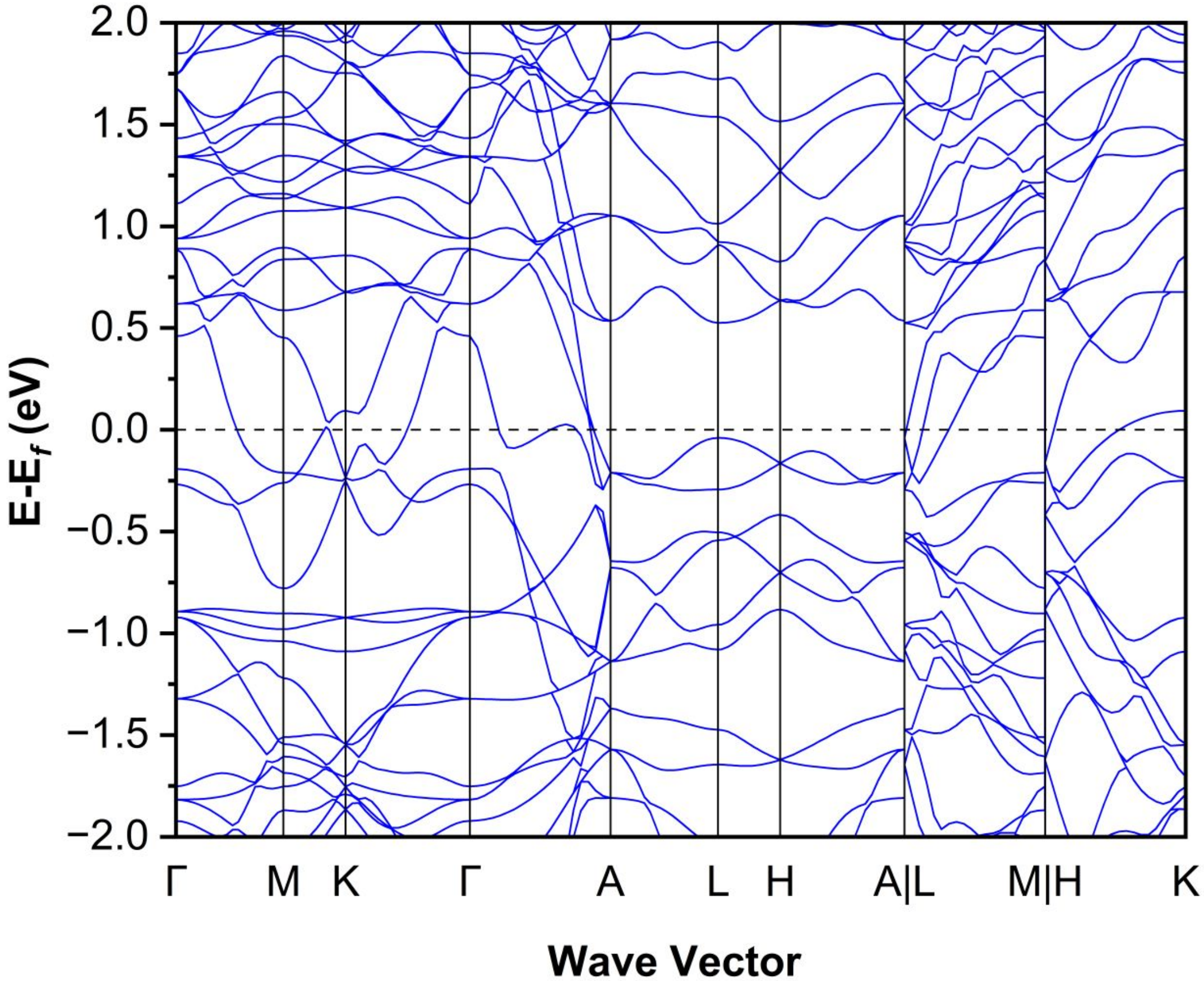

Figure S2. Band structure of Pr_3TiBi_5 without SOC.

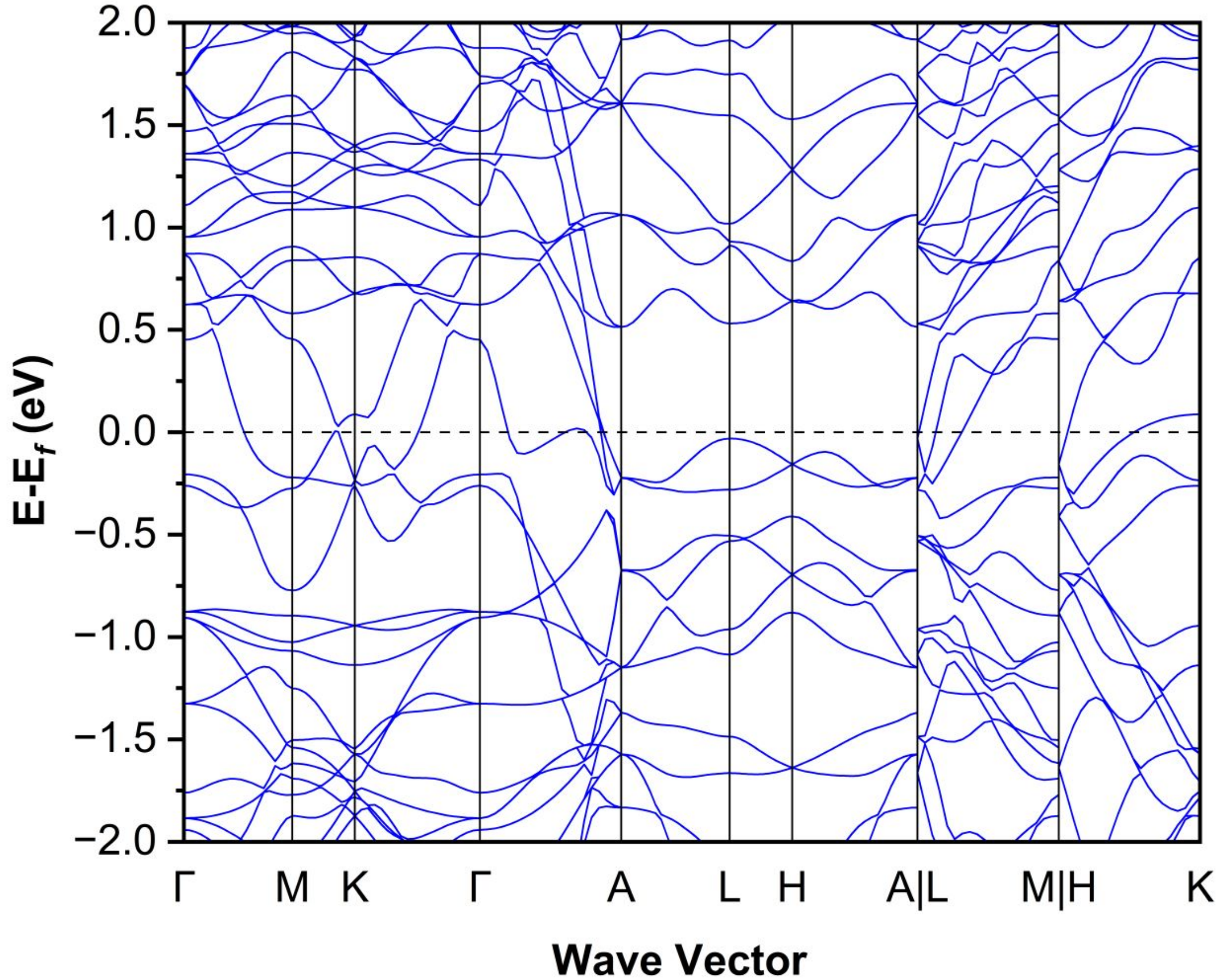

Figure S3. Band structure of Nd_3TiBi_5 without SOC.

6*g* Bi *s p*   4*d* Bi *s p*

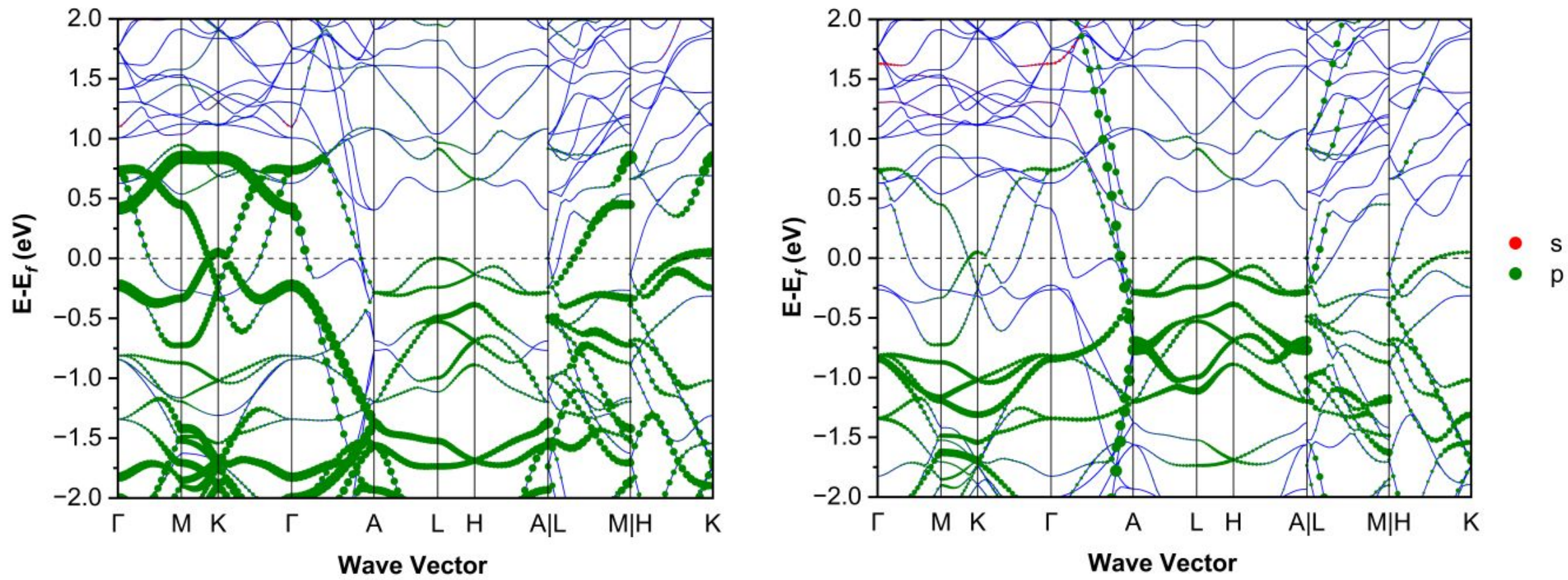

Figure S4. Orbital projected band structures for Gd_3TiBi_5 without SOC at the 6*g* and 4*d* Wyckoff positions of Bi.

$6g$ Bi p_x $4d$ Bi p_x

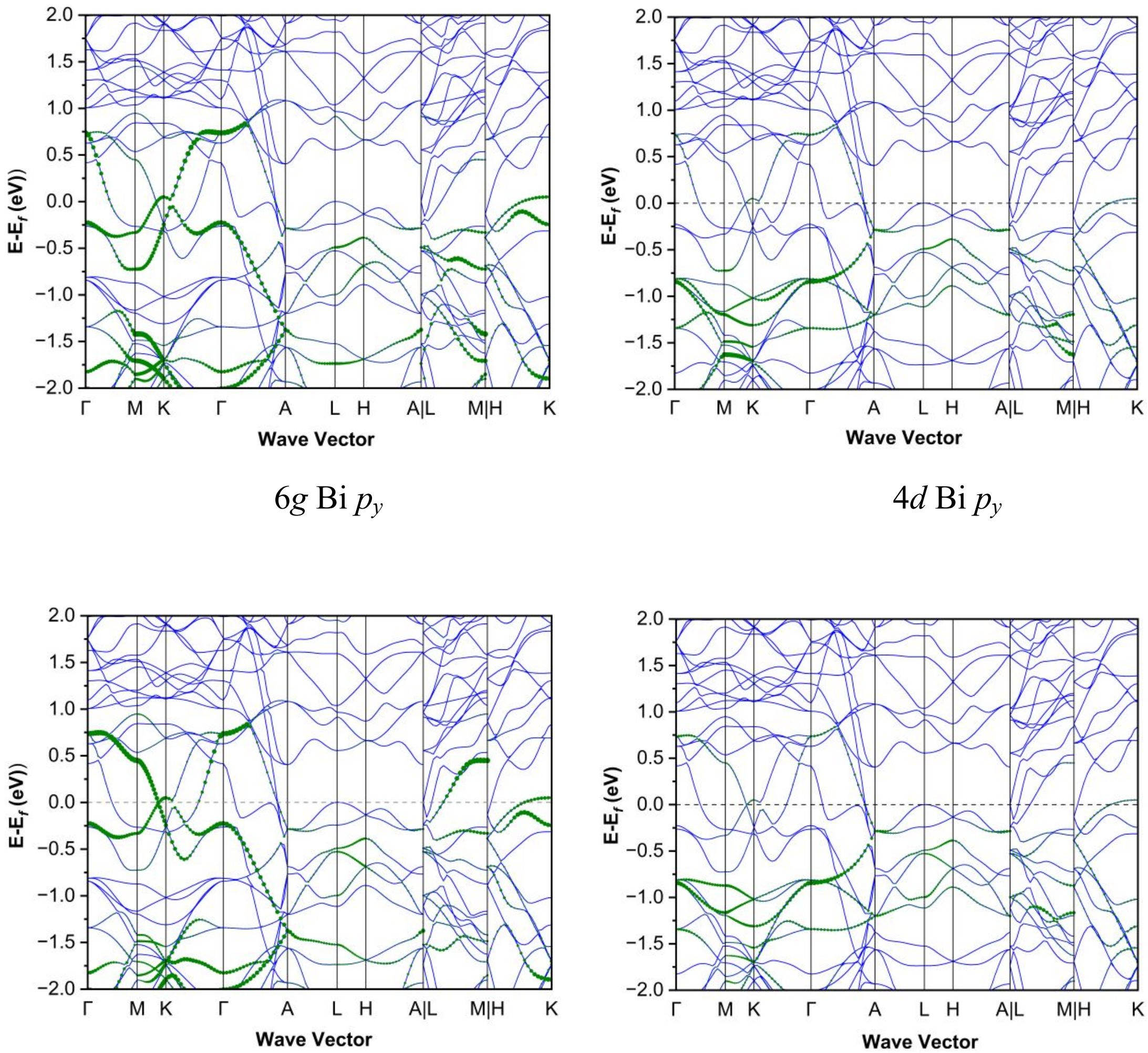

Figure S5. Projected band structures of p_x and p_y orbitals for Gd_3TiBi_5 without SOC at the $6g$ and $4d$ Wyckoff positions of Bi.

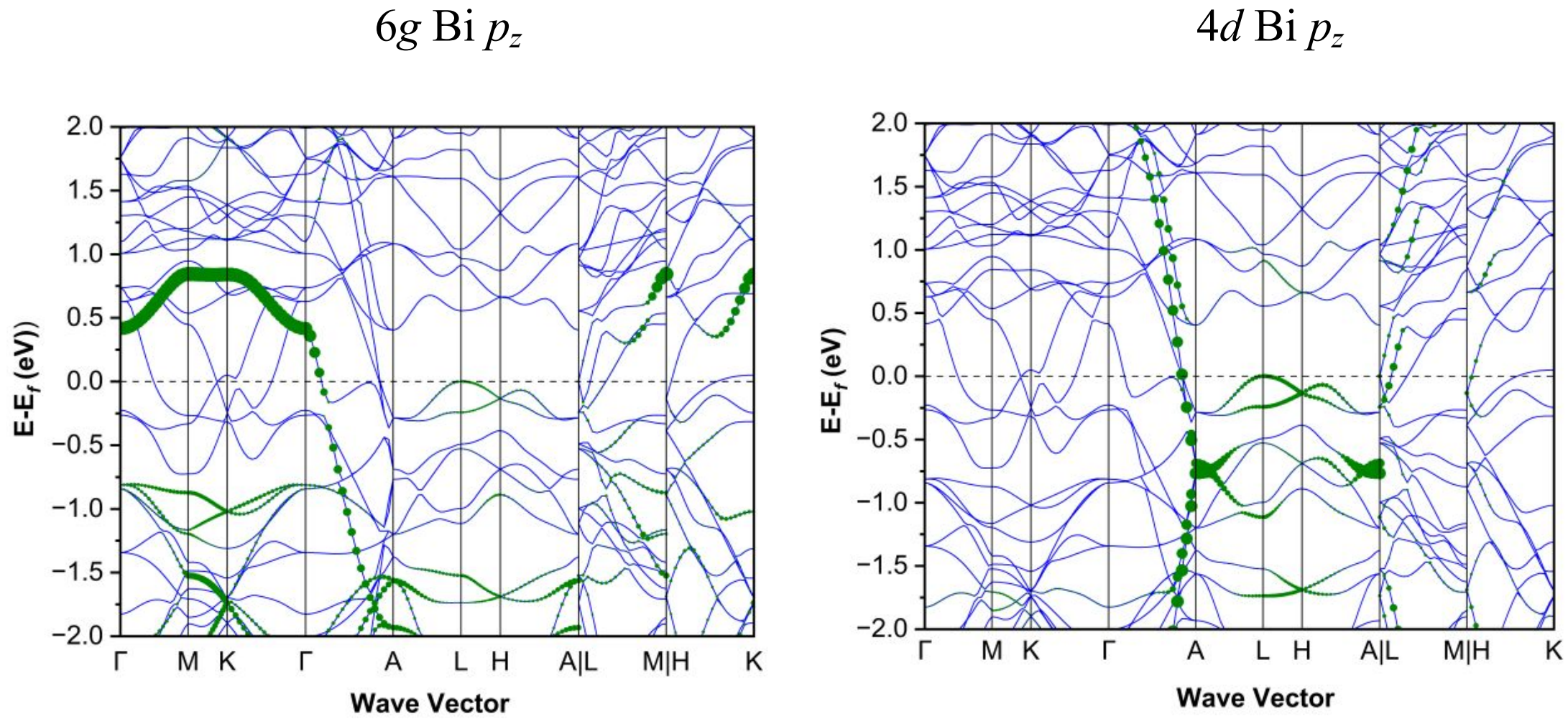

Figure S6. Projected band structures of p_z orbital for Gd_3TiBi_5 without SOC at the 6*g* and 4*d* Wyckoff positions of Bi.

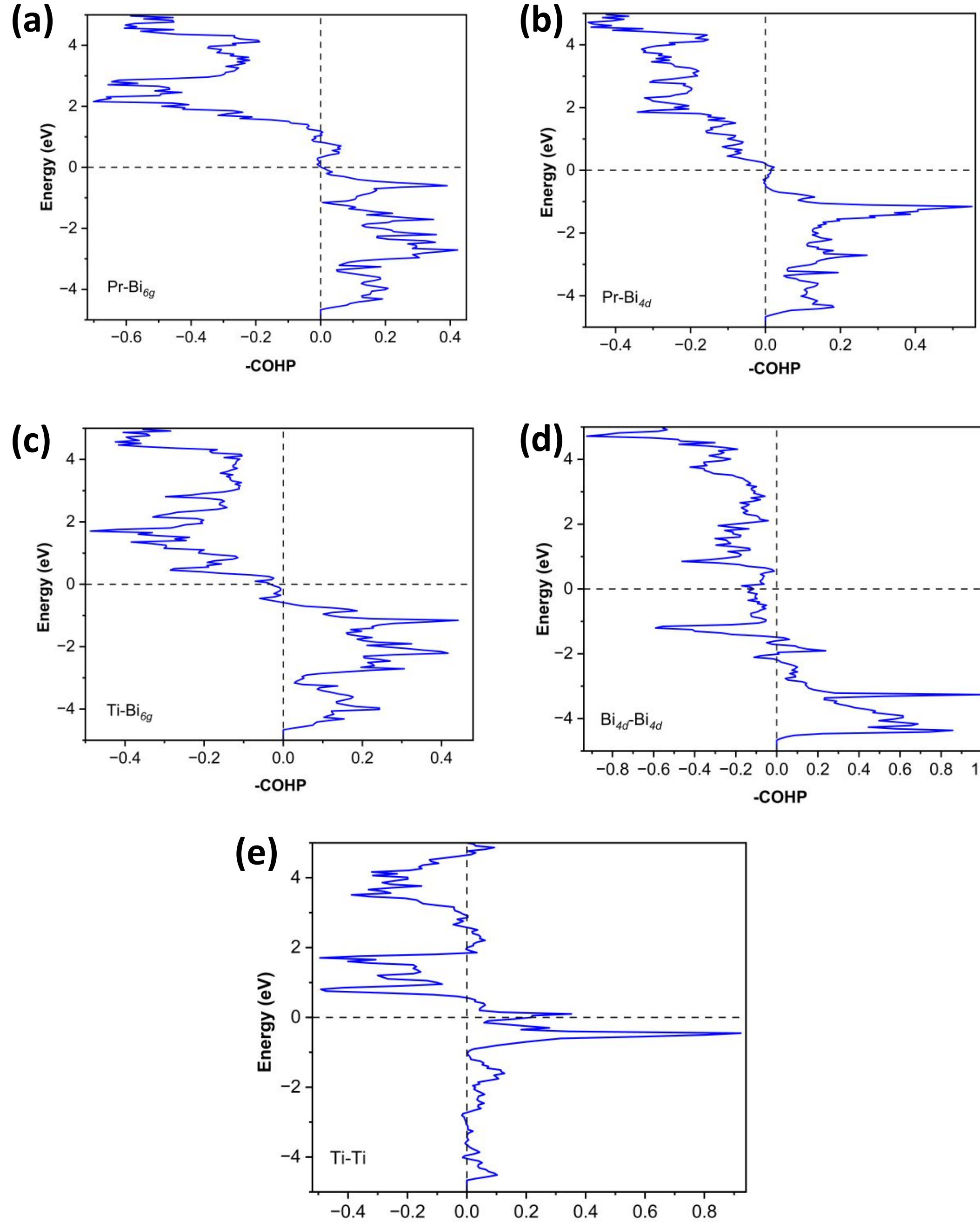

Figure S7. COHP curves in Pr_3TiBi_5. (a) Pr-Bi$_{6g}$ (b) Pr-Bi$_{4d}$ (c) Ti-Bi$_{6g}$ (d) Bi$_{4d}$-Bi$_{4d}$ (e) Ti-Ti.

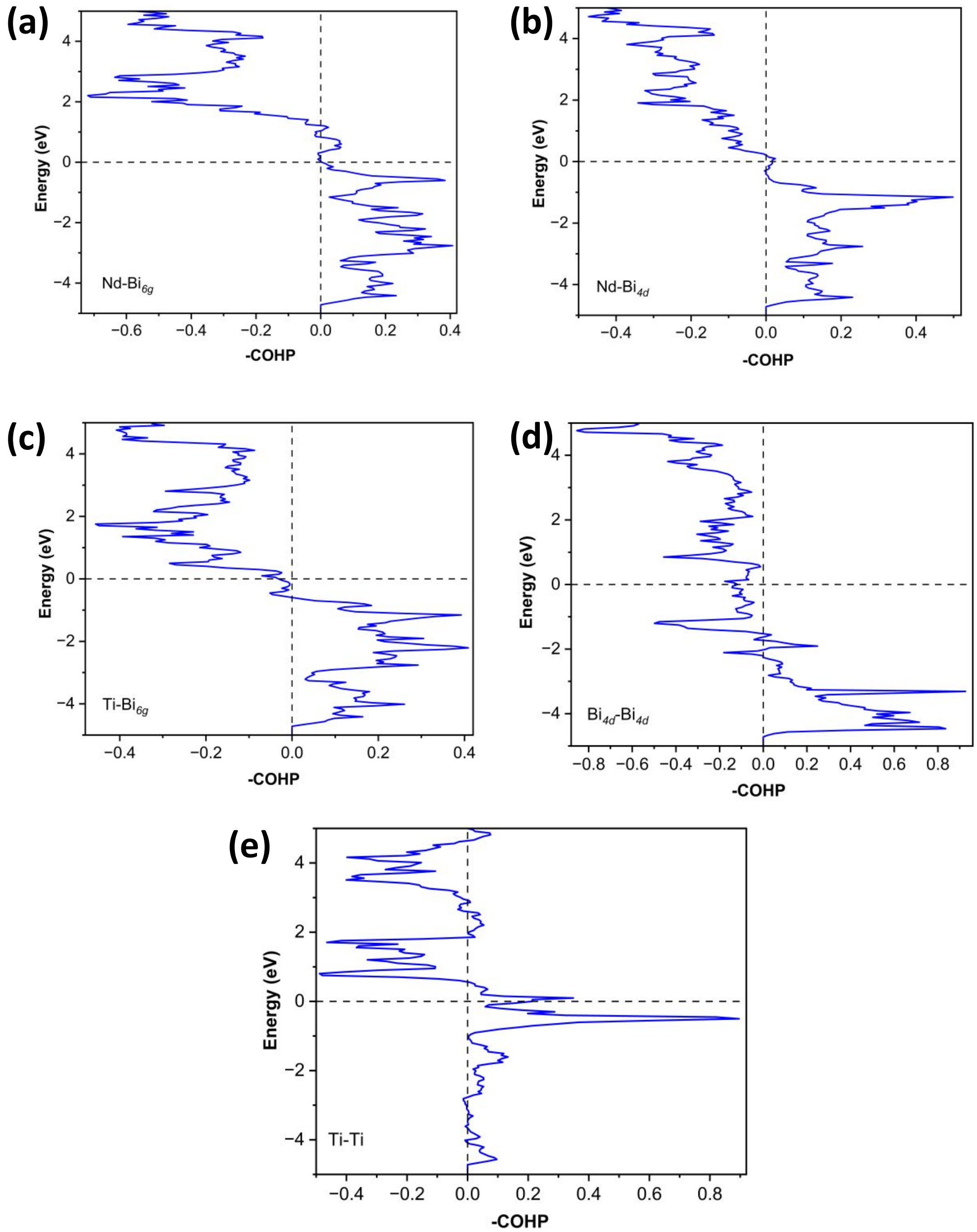

Figure S8. COHP curves in Nd_3TiBi_5. (a) Nd-Bi_{6g} (b) Nd-Bi_{4d} (c) Ti-Bi_{6g} (d) Bi_{4d}-Bi_{4d} (e) Ti-Ti.

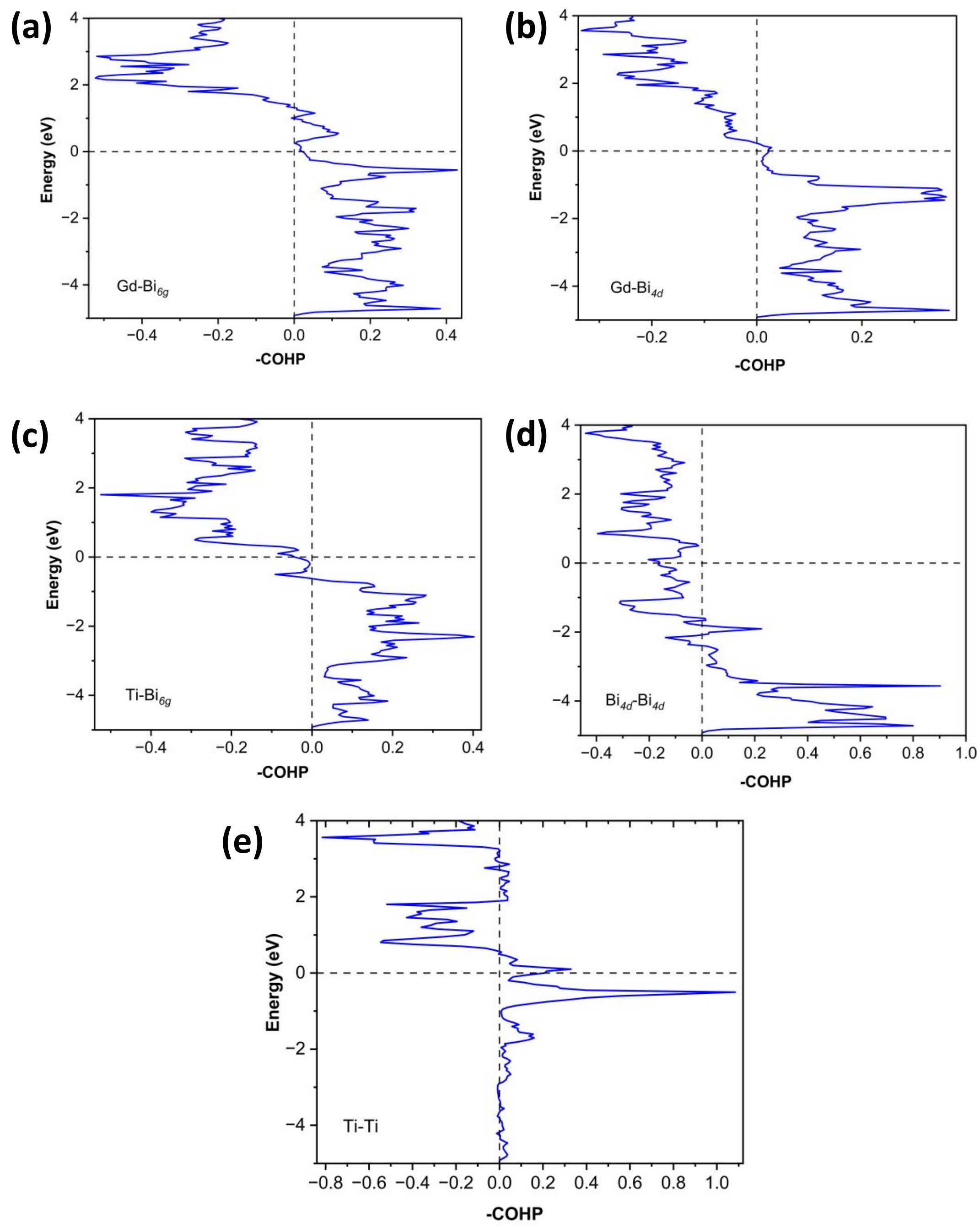

Figure S9. COHP curves in Gd_3TiBi_5. (a) Gd-Bi_{6g} (b) Gd-Bi_{4d} (c) Ti-Bi_{6g} (d) Bi_{4d}-Bi_{4d} (e) Ti-Ti.

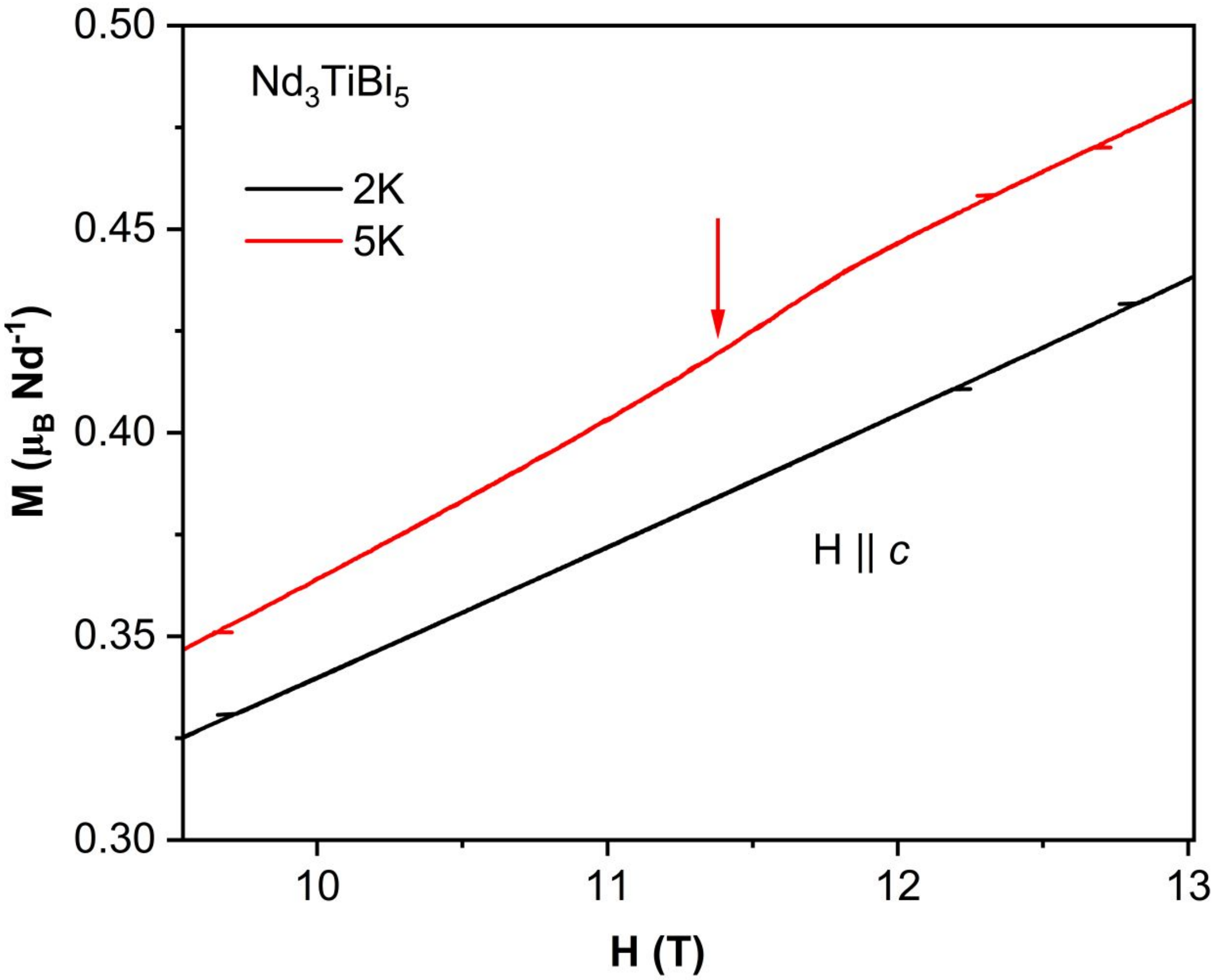

Figure S10. Magnetization vs magnetic field (9-13 T) plots of Nd_3TiBi_5 at 2 and 5K.

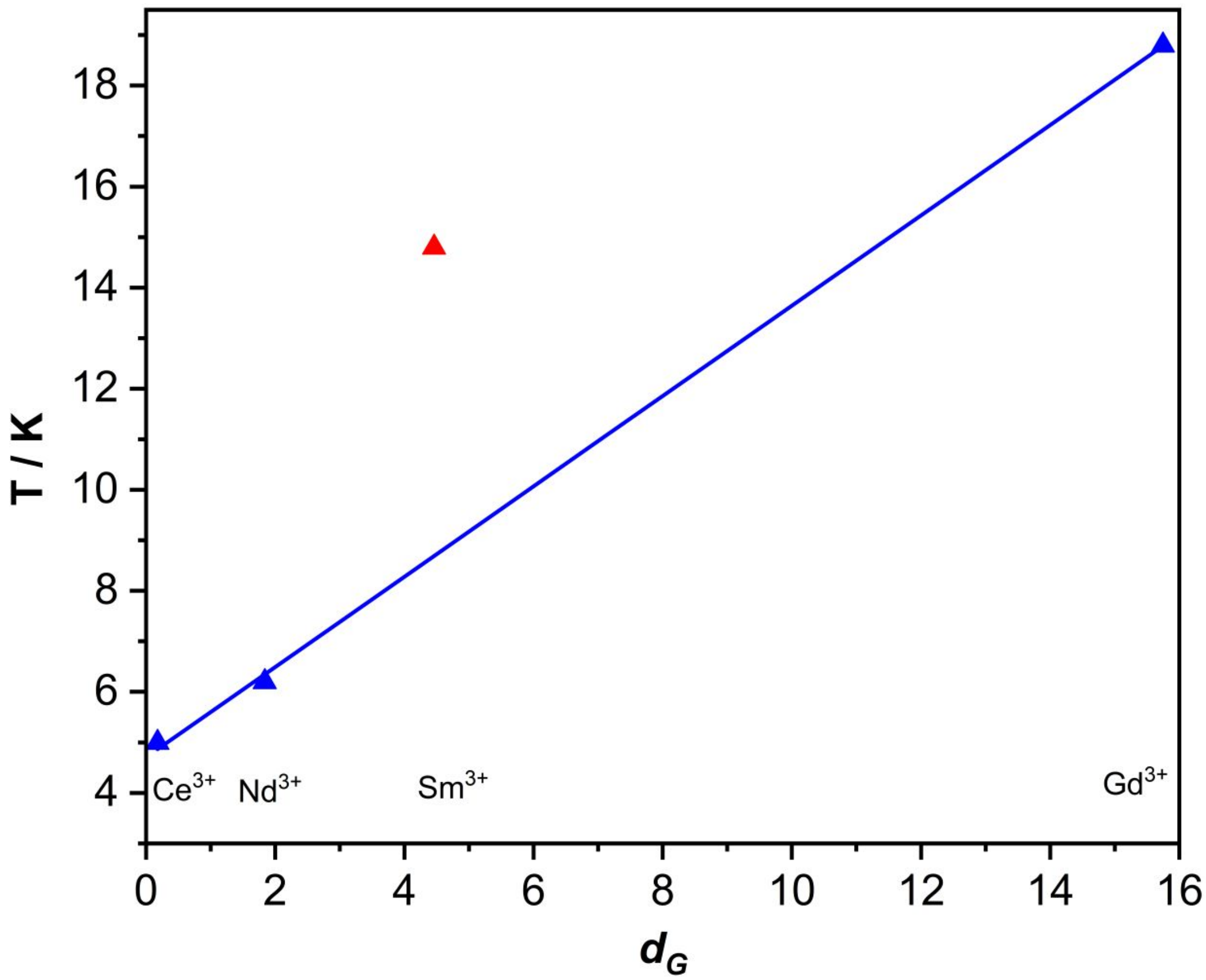

Figure S11. Magnetic transition temperatures vs de Gennes factors in Ln_3TiBi_5 (*Ln* = Ce, Nd, Sm, Gd)

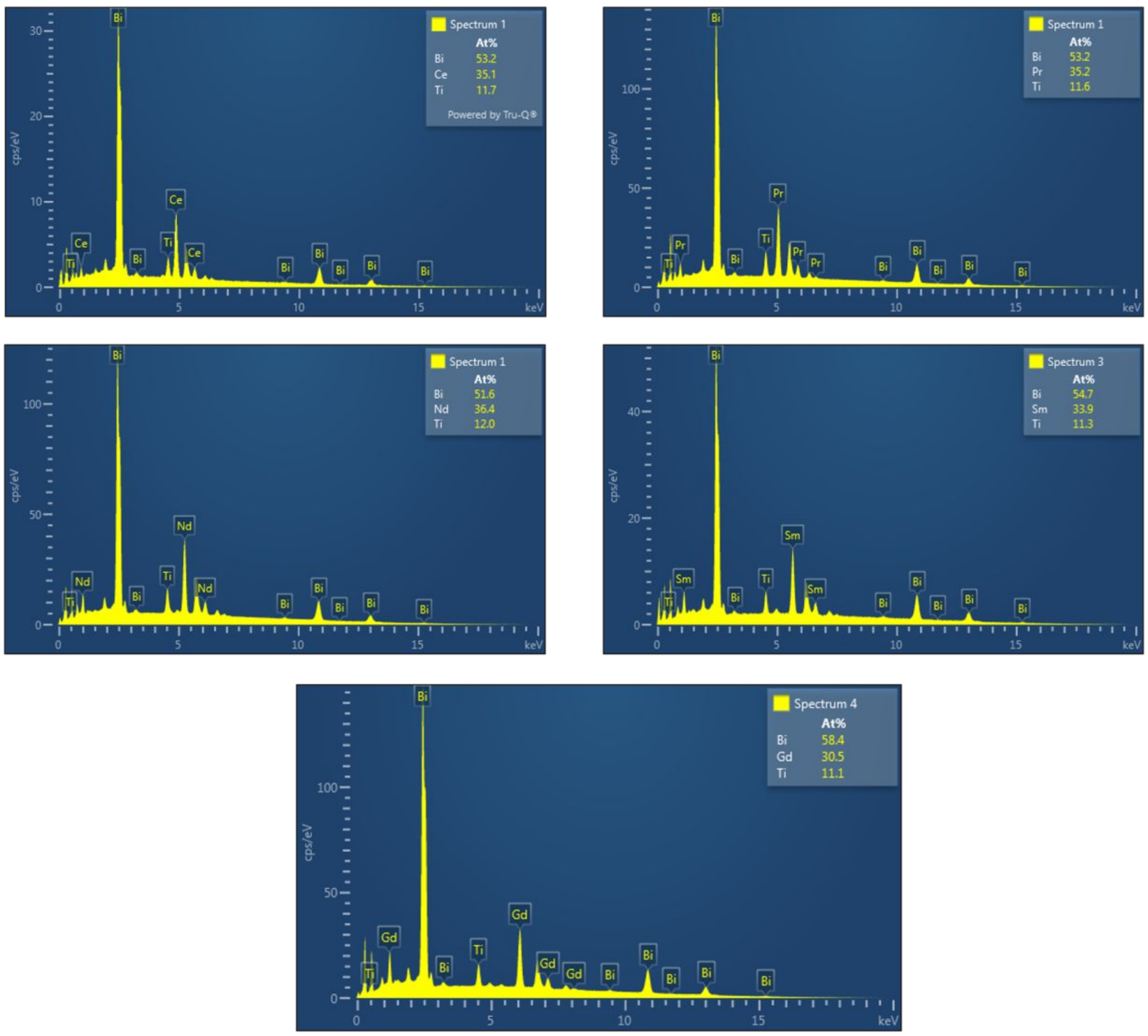

Figure S12. EDS spectra of Ln_3TiBi_5 (Ln = Ce, Pr, Nd, Sm, Gd)

Reference

1. Da-Chun, P.; Zhong-Ming, S.; Xiao-Wu, L.; Jiang-Gao, M., Synthesis and crystal structure of La_3ScBi_5. *Chin. J. Inorg. Chem.* **2006,** *22* (8), 1449-1452.

2. Xu, Z.; Wang, Y.; Zhang, C.; Liu, H.; Han, X.; Wu, L.; Zhang, X.; Wu, Q.; Shi, Y., Magnetization plateau and anisotropic magnetoresistance in the frustrated Kondo-lattice compound Ce_3ScBi_5. *Phys. Rev. B* **2024,** *110* (16), 165106.

3. Gornicka, K.; Ortiz, B. R.; Zhang, H.; Christianson, A. D.; May, A. F., Anisotropic magnetic behavior of Nd_3ScBi_5 and Pr_3ScBi_5 single crystals. *Phys. Rev. Mater.* **2025,** *9* (6), 064414.

4. Murakami, T.; Yamamoto, T.; Takeiri, F.; Nakano, K.; Kageyama, H., Hypervalent bismuthides La_3MBi$_5$ (*M*= Ti, Zr, Hf) and related antimonides: absence of superconductivity. *Inorg. Chem.* **2017,** *56* (9), 5041-5045.

5. Zelinska, O. Y.; Mar, A., Ternary rare-earth manganese bismuthides: structures and physical properties of *RE*$_3$MnBi$_5$ (*RE*= La−Nd) and $Sm_2Mn_3Bi_6$. *Inorg. Chem.* **2008,** *47* (1), 297-305.

6. Khoury, J. F.; Song, X.; Schoop, L. M., *Ln*$_3$*M*Bi$_5$ (*Ln*= Pr, Nd, Sm; *M*= Zr, Hf): Intermetallics with Hypervalent Bismuth Chains. *Z. Anorg. Allg. Chem.* **2022,** *648* (15), e202200123.

7. Khoury, J. F.; Han, B.; Jovanovic, M.; Singha, R.; Song, X.; Queiroz, R.; Ong, N.-P.; Schoop, L. M., A class of magnetic topological material candidates with hypervalent Bi chains. *J. Am. Chem. Soc.* **2022,** *144* (22), 9785-9796.

8. Pan, D.-C.; Sun, Z.-M.; Mao, J.-G., Synthesis and crystal structures of La_3MgBi_5 and $LaLiBi_2$. *J. Solid State Chem.* **2006,** *179* (4), 1016-1021.

9. Bollore, G.; Ferguson, M. J.; Hushagen, R. W.; Mar, A., New ternary rare-earth transition-metal antimonides *RE*$_3$*M*Sb$_5$ (*RE*= La, Ce, Pr, Nd, Sm; *M*= Ti, Zr, Hf, Nb). *Chem. Mat.* **1995,** *7* (12), 2229-2231.

10. Motoyama, G.; Sezaki, M.; Gouchi, J.; Miyoshi, K.; Nishigori, S.; Mutou, T.; Fujiwara, K.; Uwatoko, Y., Magnetic properties of new antiferromagnetic heavy-fermion compounds, Ce_3TiBi_5 and $CeTi_3Bi_4$. *Physica B* **2018,** *536*, 142-144.

11. Shinozaki, M.; Tsubouchi, G. M. S. N. M.; Fujiwara, K. M. K.; Manago, M., Single Crystal Growth and Physical Property Measurements of Sm_3TiX_5 (X= Bi, Sb). *International Conference on Strongly Correlated Electron Systems* **2022**.

12. Wang, R.; Zhao, Z.; Yi, X.; Zhang, Y.; Zhu, K.; Liu, H.; Lv, S.; Bai, C.; Cao, G.; Bao, L., Snowflake-like sixfold angle-dependent magnetoresistance in the kagome antiferromagnet Gd_3TiBi_5. *Phys. Rev. B* **2026,** *114* (3), 034417.
